# Electronic Conductance and Thermopower of Cross-Conjugated and Skipped-Conjugated Molecules in Single-Molecule Junctions

**DOI:** 10.1021/acs.jpcc.3c00742

**Published:** 2023-07-06

**Authors:** Rebecca J. Salthouse, Juan Hurtado-Gallego, Iain M. Grace, Ross Davidson, Ohud Alshammari, Nicolás Agraït, Colin J. Lambert, Martin R. Bryce

**Affiliations:** † Department of Chemistry, Durham University, Durham DH1 3LE, U.K.; ‡ Departamento de Física de la Materia Condensada, Universidad Autónoma de Madrid, Madrid E-28049, Spain; § Physics Department, Lancaster University, Lancaster LA1 4YB, U.K.; ∥ Condensed Matter Physics Center (IFIMAC) and Instituto Universitatio de Ciencia de Materiales “Nicolás Cabrera” (INC), Universidad Autónoma de Madrid, Madrid E-28049, Spain; ⊥ Instituto Madrileño de Estudios Avanzados en Nanociencia IMDEA-Nanociencia, Madrid E-28049, Spain

## Abstract

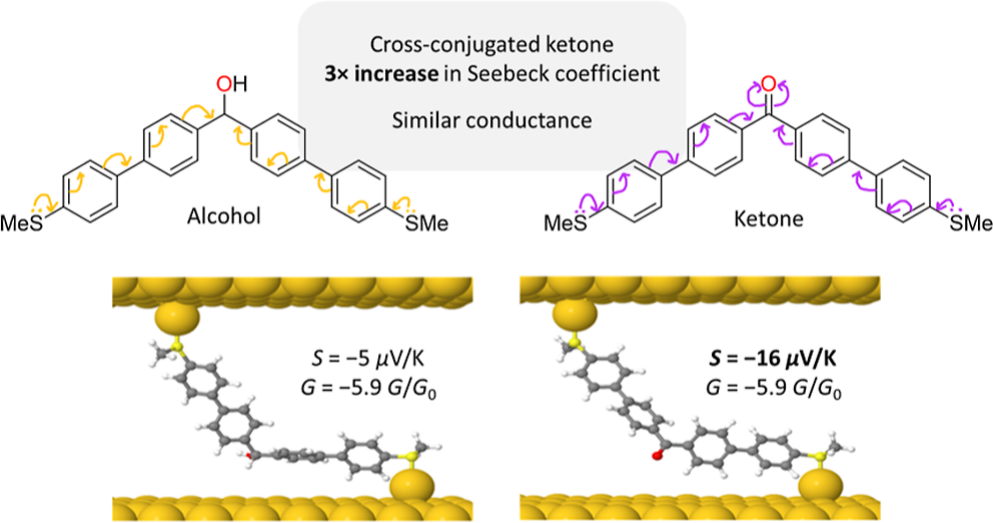

We report a combined experimental and theoretical study of a series of thiomethyl (SMe) anchored cross-conjugated molecules featuring an acyclic central bridging ketone and their analogous skipped-conjugated alcohol derivatives. Studies of these molecules in a gold|single-molecule|gold junction using scanning tunneling microscopy-break junction techniques reveal a similar conductance (*G*) value for both the cross-conjugated molecules and their skipped-conjugated partners. Theoretical studies based on density functional theory of the molecules in their optimum geometries in the junction reveal the reason for this similarity in conductance, as the predicted conductance for the alcohol series of compounds varies more with the tilt angle. Thermopower measurements reveal a higher Seebeck coefficient (*S*) for the cross-conjugated ketone molecules relative to the alcohol derivatives, with a particularly high *S* for the biphenyl derivative **3a** (−15.6 μV/K), an increase of threefold compared to its alcohol analog. The predicted behavior of the quantum interference (QI) in this series of cross-conjugated molecules is found to be constructive, though the appearance of a destructive QI feature for **3a** is due to the degeneracy of the HOMO orbital and may explain the enhancement of the value of *S* for this molecule.

## Introduction

Molecular-scale electronics continues to progress rapidly as a result of combined experimental and theoretical studies of charge transport through single-molecules that are anchored between two metallic electrodes in a molecular junction.^1−5^ A long-standing goal of this research field is to overcome the technology limits of Moore’s law^6^ by downsizing integrated circuits to the scale of a few nanometers, thereby paving the way for implementing molecular components in information technologies.^7−10^ While most studies of molecular electronics still concern fundamental science, there is the prospect of future applications of molecules in nanoscale devices such as switches, diodes, transistors, sensors, logic gates, and thermoelectric generators.^11−13^

In the context of molecular design, it is essential to understand the subtle interplay of steric and electronic factors that regulate charge transport through a molecular junction. Critical features are the charge distribution along the backbone of the molecule, the energies of frontier orbitals and the nature of the interface of the molecule with the electrodes.^14,15^ The transport properties in molecular junctions are intimately linked with resonances associated with quantum interference (QI), which can lead to enhanced (constructive CQI) or suppressed (destructive DQI) electrical conductance.^16−23^ The majority of molecules studied in single-molecule junctions possess highly conjugated backbones, for example oligo(arylene-ethynylene)s^24,25^ or oligo(arylene)s,^26^ with para-connectivity between the monomer units to maximize the extent of linear π–electron conjugation and thereby enhance electrical conductance through CQI.

Alongside studies of conductance, research into the thermoelectric properties of organic materials is rapidly expanding, due to their potential applications as flexible thermogenerators and Peltier coolers. Single-molecule junctions are an ideal test-bed for probing structure-thermoelectric property relationships.^27,28^ An efficient thermoelectric material should have a high Seebeck coefficient *S*, high electrical conductance *G,* and low thermal conductivity κ. DQI in a junction creates an anti-resonance close to the Fermi energy (*E*_F_), leading to an increase in the value of *S* but reducing the value of *G*. DQI has been shown to be a prominent feature of meta-linked anchoring groups and backbones.^22,29,30^ Cross-conjugation is an important concept^31^ that has led to interesting conductance and QI effects that have been predicted theoretically and observed experimentally in some cases.^32−37^ However, there remains considerable scope to explore new molecules with different conjugation pathways, especially their thermopower properties.

**Figure 1:**
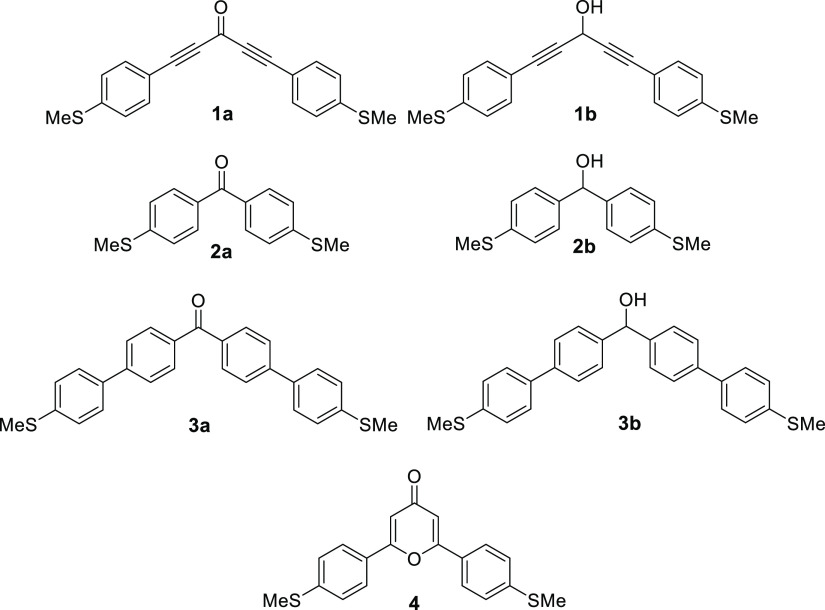
Structures of compounds used in this study bearing cross-conjugated ketone groups (**1a–3a** and **4**) and the analogous skipped-conjugated alcohol derivatives (**1b**–**3b**).

We now report on a series of cross-conjugated molecules **1a–3a** and **4** with a central bridging ketone group in the backbone, and the skipped-conjugated (broken-conjugated) alcohol analogs **1b–3b** (Figure 1). Measurements of electrical conductance and Seebeck coefficient using scanning tunneling microscopy-break junction (STM-BJ) techniques, combined with density functional theory (DFT) calculations, establish some key structural features of these types of molecules that determine the conductance and the thermopower of the single-molecule junctions.

## Results and Discussion

### Molecular Design and Synthesis

Cyclic ketones, such as anthraquinone,^21,38−40^ fluorenone,^30,35,41,42^ and diketopyrrolopyrrole^43−45^ derivatives, have been studied in detail in molecular junctions. However, we are not aware of any comparable studies on the transmission properties (conductance or thermopower) of molecules with an acyclic ketone or a pendant alcohol group in the backbone. These acyclic structures are of special interest because, unlike the cyclic ketones, with the exception of anthraquinone when anchors are attached to different phenyl rings (2,6- or 2,7-di-substitution), transmission must pass through the sp^2^ or sp^3^ carbon atom of the R_2_C=O or the R_2_CH(OH) unit, respectively, at the core of the molecule. This may lead to interesting QI features that are not present in the cyclic ketone analogs where an alternative pathway through a C–C bond is available. Additionally, conformational flexibility could play a role in the acyclic molecules. The present work concerns molecules **1–4** which have thiomethyl groups attached at both termini to assemble gold|single-molecule|gold junctions. The detailed procedures for the synthesis of **1–4** are reported in the Supporting Information, along with their characterization by NMR spectroscopy, mass spectrometry, and elemental analysis, including the single-crystal X-ray structure of **4**.

Our choice of target molecules proceeded as follows. Based on our recent study of cross-conjugated enediyne derivatives,^37^ we initially studied diynones derivative **1a** and were surprised that upon attempted assembly of **1a** in the junction, no conductance peak could be observed within the range of our experimental setup (i.e., *G* < ≈10^–6.5^*G*_0_). This result is in contrast with the expected observable conductance based on theoretical calculations for **1a** (see below). A possible explanation for this discrepancy is that **1a** rearranges or degrades on the gold surface. Indeed, while our work was in progress, it was reported that related diynones rearrange to γ-pyrones in the presence of gold nanoparticles supported on titania (Au/TiO_2_) in solution, initiated by the aurophilicity of the triple bonds.^46^ We, therefore, synthesized the corresponding γ-pyrone derivative **4** by acid-catalyzed rearrangement of **1a** following a literature route:^47^ compound **4** would be the expected product of the Au-catalyzed rearrangement of **1a**. To our surprise, no conductance could be measured for **4** in junction experiments, in disagreement with the theory for **4** (see below). We next synthesized diaryl ketones **2a** and **3a** which do not have any alkyne units. Analogous alcohol derivatives of **1a**, **2a,** and **3a** (namely, **1b–3b**) were also synthesized to explore the effect of cross-conjugated ketone versus skipped-conjugated alcohol functionality at the core of the molecules. The conductance and thermopower of **2a**, **3a,** and **1b–3b** were successfully measured and the experimental data are discussed below, supported by quantum transport calculations.

### Conductance (*G*) and Thermopower (*S*) Measurements

*G* measurements of molecular junctions were performed using a modified home-built scanning tunneling microscope (STM) at ambient conditions and room temperature using the STM-break junction (STM-BJ) technique.^48^ The sample is contacted with the STM tip and, when the tip is retracted, atomic Au contacts form before the metallic contact breaks and a molecule may be trapped between both electrodes, forming a gold|single-molecule|gold junction. By recording the current during this whole process, current-distance (*IZ*) curves are obtained, showing a plateau in *G* values as a signature of molecular junction formation.

**Figure 2:**
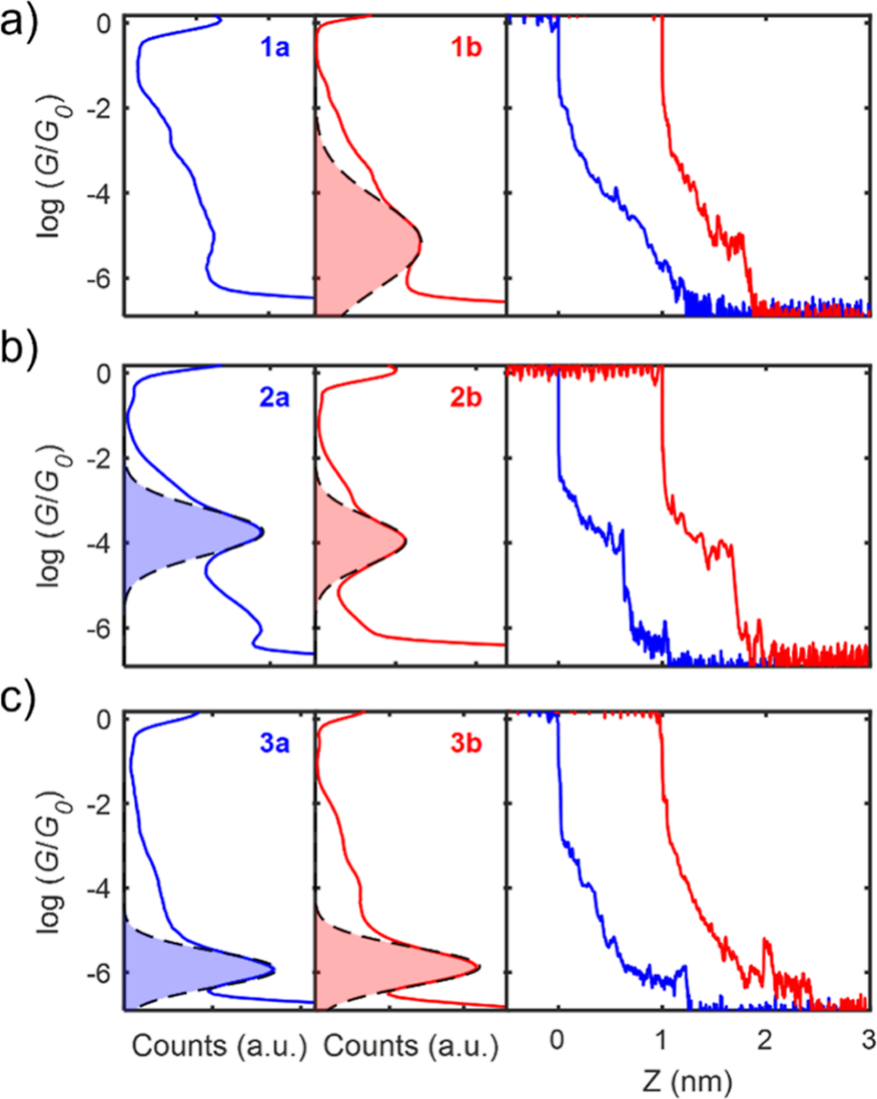
Conductance measurements of compounds **1–3**. Left panels show 1D conductance histograms (blue lines) and Gaussian fit to the conductance peak (black line and blue filled area) of compounds **1a** (a), **2a** (b), and **3a** (c). Middle panel shows 1D conductance histograms (red lines) and Gaussian fit to the conductance peaks (black dashed line and filled red area) of compounds **1b** (a), **2b** (b), and **3b** (c). Right panel shows individual current vs distance traces of the acyclic ketone (blue line) and pendant alcohol compounds (red line) of compounds **1** (a), **2** (b), and **3** (c).

1D conductance histograms of compounds **1–3**, shown in Figure 2, were performed by collecting thousands of *IZ* traces and eliminating those without a clear molecular plateau using a non-supervized clustering technique.^49,50^ In the case of compound **1a**, no clear plateau signature was found in the *IZ* traces; therefore, all traces are included in the 1D conductance histogram, shown in the left panel of Figure 2a. Gaussian distributions were fitted to all the main conductance peaks, as shown in the left and middle panels of Figure 2, and mean conductance values, shown in Table 1, were obtained from their mean value. No conductance was observed for compound **4**, contrary to theory (see Figure S18). We tentatively propose this molecule is decomposing during the measurements; there is precedent for nucleophilic attack at a carbon adjacent to the cyclic oxygen in solution experiments.^51,52^

**Table 1:** (a) Experimental STM-BJ Results of all Compounds^a^

			**1a**	**1b**	**2a**	**2b**	**3a**	**3b**	**4**
(a)	experimental	*L*_*s*_ (nm)	×	0.9	0.8	0.8	1.5	1.5	×
		*G* log (*G*/*G*_0_)	×	–5.0	–3.7	–4.0	–5.9	–5.9	×
		*S* (μV/K)	×	–2.6	–3.5	–2.5	–15.6	–5.0	×
	theoretical	*L* (nm)	1.5	1.5	1.1	1.1	1.8	1.8	1.2
(b)		*G* log (*G*/*G*_0_)	–4.1	–5.7	–4.7	–4.9	–6.4	–5.8	–6.5
		*S* (μV/K)	–21.9	–7.9	–17.4	–8.3	–24.0	–1.1	–19.9

a The crosses indicate that no significant conductance plateau was observed. *L*_*s*_ is the apparent stretching length (see Supporting Information for details). (b) Theoretical results of all compounds evaluated for the optimum geometry at tilt angle θ = 60°.

Similar conductance values were obtained for the acyclic ketones and their analogous compound with a pendant alcohol group, with a conductance value of around *G* ≈ 10^–4^*G*_0_ for compounds **2a–b** and *G* ≈ 10^–6^*G*_0_ for compounds **3a–b**. These results highlight that cross-conjugation versus skipped-conjugation has no significant influence on the conductance of these molecules. Individual *IZ* traces of compounds **1–3** are shown in the right panels of Figure 2 and clear conductance plateaus are observed in all compounds except for compound **1a**, in contrast to the cross-conjugated alkene analogs studied previously.^37^ The apparent stretching length (*L*_s_) was measured for all the conducting compounds (more information about the procedure is provided in the Supporting Information), showing that, as expected, plateau lengths for each group of compounds with different pendant groups are the same.

Comparing these results to reported cyclic derivatives, cross-conjugated anthraquinone is known to be less conductive than its reduced linear conjugated radical anion,^38,39^ as well as anthracene and the broken-conjugated dihydroanthracene,^21^ due to a prominent DQI feature close to the electrode Fermi energy for anthraquinone. Reported conductance values for reduced anthraquinone (10^–4.6^*G*_0_ to 10^–5.4^*G*_0_) are within a similar range to the results presented here for both the alcohol and ketone derivatives. In addition, similar conductance values have been reported for para-connected fluorene/fluorenone molecules. However, meta connectivity gave a much lower conductance value for fluorene compared to the analogous cross-conjugated fluorenone due to almost entire suppression of DQI.^35^

To measure *S*, a temperature difference between the tip and the sample is created by heating up the tip with a 1 kΩ series resistor placed on the tip holder. The tip temperature, *T*_h_, increases as the sample temperature, *T*_c_, remains at room temperature. These temperatures were measured using thermocouples placed on top of the resistor and sample, respectively. The thermovoltage response of the molecule (*V*_th_) in the junction is measured by performing small current–voltage (*IV*) ramps of ±10 mV while the molecule is in the junction. By calculating their zero-current crossing point and slope, *V*_th_ and *G* are simultaneously measured, respectively. As the Seebeck coefficient is defined by *S* = −Δ*V*/Δ*T*, various temperatures are applied to the tip in order to calculate *S* by performing a linear regression of all the measured *V*_th_ values vs Δ*T* points, where Δ*T* = *T*_h_ – *T*_c_.

**Figure 3:**
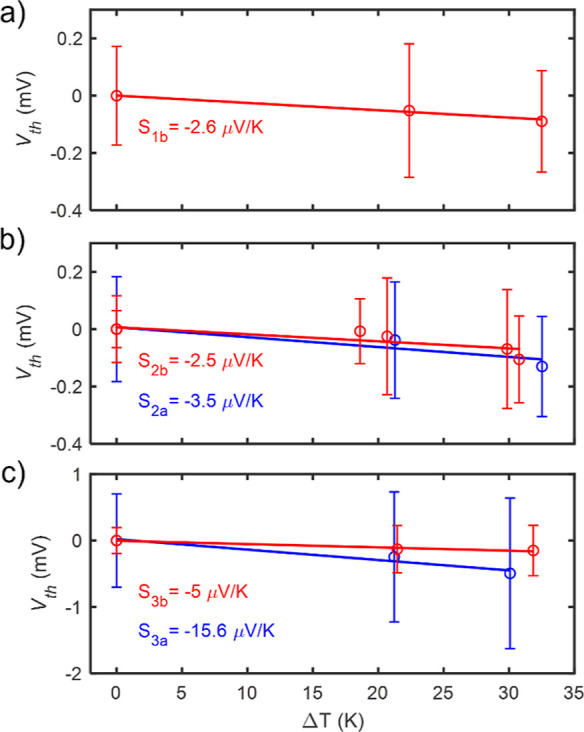
Seebeck coefficient measurements of compounds **1b** (a), **2** (b), and **3** (c). Linear regression of all the measured *V*_th_ vs Δ*T* points for each molecule are shown as solid lines. Empty circles and error bars are the mean values and standard deviations of each individual measurement, respectively. Blue lines represent the acyclic ketone compounds and red lines the corresponding compounds with a pendant alcohol group.

Figure 3 shows, as solid lines, the linear regressions of compounds **1b**, **2,** and **3**, where empty circles and error bars represent the mean value and standard deviation of all the different *V*_th_ vs Δ*T* measured sets, respectively. The Seebeck coefficients of molecules **2** and **3** indicate that the presence of a pendant acyclic ketone (blue lines) increases *S* with respect to the analogous compounds bearing a pendant alcohol group. In particular, there is a notable increase in *S* for compound **3a** compared to **3b**, with values of −15.6 and −5 μV/K, respectively. The negative sign of *S* for all molecules is a signature of LUMO-dominated electronic transport, as previously reported for SMe anchor groups.^53^ The experimental STM-BJ data are presented in Table 1, in comparison with values obtained by theoretical calculations (see below).

### Theoretical Calculations

To gain further insight into the structure-transmission property relationships in molecules **1–4**, quantum transport calculations were performed. The density functional code SIESTA^54^ and transport code GOLLUM^55^ were used to calculate the conductance and Seebeck coefficients of molecules **1–4**. First the optimum geometry of each molecule was found and then the molecule was attached to gold electrodes. Our previous work^37^ investigated the importance of the molecular orientation in determining the behavior of cross-conjugated molecules. Here we consider two configurations, determined by the tilt angle, θ, where θ is the angle between the axis of the molecule (defined by the terminating sulfur atoms) and the normal of the gold surface (Figure S37).

**Figure 4:**
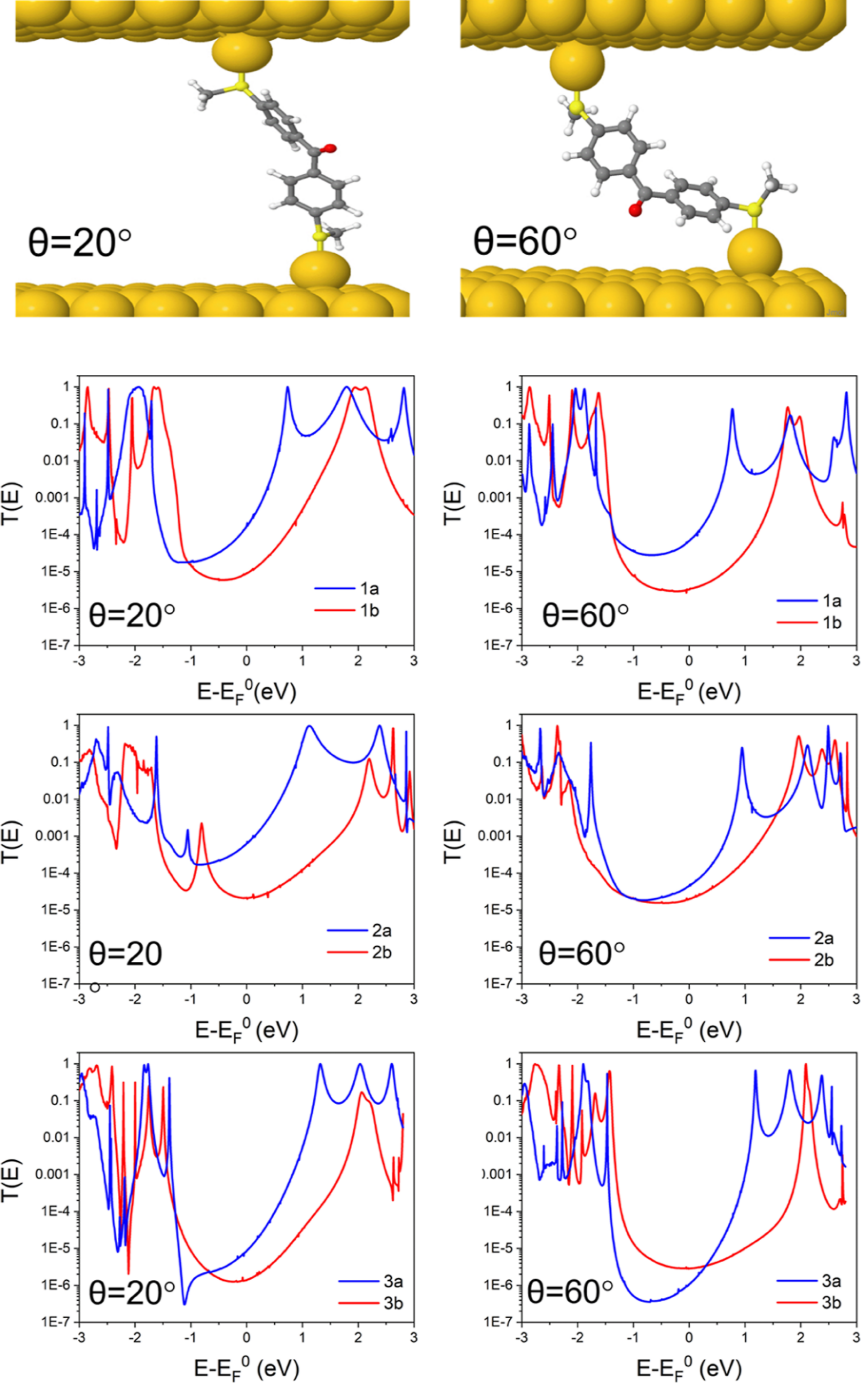
(Top) Junction geometry for molecule **2a** connected to gold electrodes via a gold adatom for a tilt angle of 20° (left) and 60° (right). Zero bias transmission coefficient, *T*(*E*), versus electron energy, *E*, for molecules **1a** vs **1b**, **2a** vs **2b,** and **3a** vs **3b** at θ = 20° (left) and θ = 60° (right).

The SMe anchor groups attach to a surface adatom and the optimum geometry was calculated at angles of θ = 20° and θ = 60°. Figure 4 shows example junction configurations for molecule **2a**, where θ = 60° is the optimum configuration and the smaller tilt angle is less energetically favorable. The zero bias transmission coefficient, *T*(*E*), was then calculated for each of the molecules as a function of electron energy, *E*, with the resulting data shown in Figure 4. The two angles were chosen to explore the difference between the optimum angle of 60° and the more linear junction of 20°. This shows how the transmission coefficients of cross and skipped molecules exhibit different dependencies on the tilt angle. In the case of the small tilt angle, the cross-conjugated molecules have a much larger conductance than the skipped molecules. This is to be expected, because the HOMO–LUMO gap of the cross-conjugated molecules is larger than that of the skipped molecules, in contrast with our experimental measurements. However, for the larger tilt angle, this theoretical behavior does not hold, especially for molecules **2** and **3**, where the transmission coefficients are very similar for both the cross and skipped molecules around the Fermi energy. This agrees with the experimental measurements, which show similar conductances for **2** and **3**.

The experimental stretching lengths for molecules **1a** and **1b** are 0.8 nm in contrast with the theoretical lengths of the molecules, which are 1.1 nm. For **2a** and **2b,** the stretching length is 1.5 nm in contrast with the theoretical molecular lengths of 1.8 nm. In both cases, the experimental stretching lengths are shorter than the lengths of the molecule, which is consistent with a tilted geometry.

It has been shown that cross-conjugated molecules demonstrate quantum interference in molecular junctions and that cross-conjugation typically leads to destructive quantum interference (DQI) features.^25^ Cyclic derivatives also have predicted QI behavior, with those containing anthraquinone units showing DQI,^56^ whereas those with fluorenone units show a Fano resonance.^41,57^ A simple method to determine the nature of the interference is the orbital product rule,^58^ which predicts that DQI will occur when the orbital products of the contact site have the same sign for the HOMO and LUMO, and constructive quantum interference (CQI) will occur when they have the opposite sign. The HOMO and LUMO orbitals for molecules **1–4** are shown in Figures S20–S26 (the sign of the wave function is denoted blue for negative and red for positive). For the cross-conjugated compounds **1a**, **2a**, **3a,** and **4**, the wavefunction on both contact atoms has the same sign for the LUMO and the opposite sign for the HOMO, indicating constructive interference. Molecules **1b**, **2b,** and **3b** are also predicted to show constructive interference.

Comparing the transport for the cross-conjugated with the skipped-conjugated molecules shows that for molecules **1a** and **1b**, the behavior in the HOMO–LUMO gap shows CQI and a significant increase in the transmission value around the Fermi energy (*E* – *E*_F_^0^ = 0 eV) (larger than an order of magnitude) for the ketone-based molecule at both tilt angles of 20 and 60°. This can be attributed to the smaller HOMO–LUMO gap of molecule **1a** in comparison with **1b** (Table S2) and the lower lying LUMO level. Again, comparing molecules **2a** and **2b**, the transmission curves both show CQI in the gap and at θ = 20°, the value for **2a** is much larger. However, for the transmission at the optimum tilt angle of 60°, there is very little difference between the two molecules, with the value close to the Fermi energy slightly larger for **2a**; this matches the trend in the measured values, as shown in Table 1. The difference in response between **1a** vs **1b** and **2a** vs **2b** can be attributed to the structure of the molecules. **1a** and **1b** are planar so the coupling between the electrodes as the molecule is tilted is consistent, whereas **2a** and **2b** are non-planar with the twisting of the rings in **2b** being larger, hence the coupling to the leads is no longer symmetric.

For molecule **3a** at θ = 20°, there is a clear DQI feature close to the HOMO resonance (*E* – *E*_F_^0^ = −1 eV), whereas the molecular orbitals of the HOMO and LUMO (Figure S24) predict CQI. However, for this molecule, the HOMO and HOMO-1 energy levels are degenerate, which may explain the deviation from the orbital product rule expectation. Comparing **3a** to **3b** at θ = 20° reveals that the transmission is higher for **3a** at the Fermi energy, but when the angle is increased to 60°, the order is reversed and the transmission for **3b** is slightly higher.

Evaluating the room temperature conductance and Seebeck coefficient at the DFT predicted Fermi energy for the optimum tilt angle of 60° gives the results shown in Table 1. The similarity in conductance values between the ketone compounds and their analogous alcohol derivatives agrees with the trend in the experimental measurements with the ordering **2b** > **1b** > **3b** and **2a** > **3a**. In terms of the Seebeck coefficient, the molecules all show negative values, with a higher magnitude of *S* for the cross-conjugated molecules compared to the skipped-conjugated, which can be attributed to the lower lying LUMO levels of the former. Molecule **3a** is predicted to have the highest value of *S*, in agreement with experiment, and while the DQI feature does not sit close to the Fermi energy for this molecule, it does play a role in enhancing the value of *S*.

## Conclusions

In summary, we have synthesized a series of compounds featuring an acyclic cross-conjugated ketone group and their analogous alcohol derivatives, all with SMe anchor groups. The difference in the conjugation of the molecules is not matched by a difference in the magnitude of the conductance, which is measured to be similar for both the cross-conjugated molecules and their skipped-conjugated partners. This is explained by considering the different orientations that these molecules take when the molecules are tilted in their optimum configuration; *G* changes more significantly with tilt angle for the alcohol derivatives **1b–3b**. We have also shown that the Seebeck coefficient is increased for the ketone derivatives. In particular, an unusually high Seebeck coefficient has been measured for molecule **3a** (*S***=** −15.6 μV/K), an increase of threefold compared to its alcohol analog. This enhancement is in qualitative agreement with previous studies of DQI in single molecules.^59−61^ The predicted behavior of the quantum interference in this series of cross-conjugated molecules is found to be constructive, in contrast to previously studied cross-conjugated alkene molecules.^37^ As shown in Figures S20–25, the HOMOs and LUMOs of all molecules have opposite symmetries, so the orbital product rule^62^ predicts that all molecules should exhibit CQI. One exception to this is **3a**, which has a degenerate HOMO and therefore the orbital product rule does not apply, which is why this molecule can exhibit a DQI feature in its transmission curve.

Overall, this study expands the range of molecules that have been assembled and characterized in single-molecule junctions. The particularly high *S* of **3a** should stimulate further studies on cross-conjugated molecules and represents a step in the development of structure-thermoelectric property relationships toward producing more efficient organic thermoelectric devices for future applications.

## Supporting Information Available

General experimental methods and spectroscopic characterization for compounds **1–4**; procedures and additional data for conductance and thermopower measurements; computational methods and results and crystallographic data for compound **4** CSD-2239000 (PDF)
