# Supplementary material for: Electronic Conductance and Thermopower of Cross-Conjugated and Skipped-Conjugated Molecules in Single-Molecule Junctions
Source: J Phys Chem C Nanomater Interfaces. 2023 Jul 6;127(28):13751–8. doi: 10.1021/acs.jpcc.3c00742 (PMC10389811; doi:10.1021/acs.jpcc.3c00742)
Supplement: Supplementary file 1 — jp3c00742_si_001.pdf [file jp3c00742_si_001.pdf]

SUPPORTING INFORMATION

Electronic Conductance and Thermopower of Cross-Conjugated and Skipped-Conjugated Molecules in Single-Molecule Junctions

Rebecca J. Salthouse,[1,‡] *Juan Hurtado-Gallego,*[2,‡] *Iain M. Grace*[3,‡] *Ross Davidson,*[1,] *Ohud Alshammari,*[3,] *Nicolás Agraït,*[2,4,5,*] *Colin J. Lambert*[3,*] *and Martin R. Bryce*[1,*]

[1]Department of Chemistry, Durham University, Durham DH1 3LE, United Kingdom

[2]Departamento de Física de la Materia Condensada, Universidad Autónoma de Madrid, E-28049 Madrid, Spain

[3]Physics Department, Lancaster University, Lancaster, LA1 4YB, United Kingdom

[4]Condensed Matter Physics Center (IFIMAC) and Instituto Universitatio de Ciencia de Materiales 'Nicolás Cabrera' (INC), Universidad Autónoma de Madrid, E-28049 Madrid, Spain

[5]Instituto Madrileño de Estudios Avanzados en Nanociencia IMDEA-Nanociencia, E-28049 Madrid, Spain

‡Authors with equal contributions.

Corresponding Authors: nicolas.agrait@uam.es; c.lambert@lancaster.ac.uk; m.r.bryce@durham.ac.uk

Keywords: Molecular electronics, scanning probe microscopy, quantum transport, conductance, organic thermoelectric, ketone, alcohol, cross-conjugation, skipped-conjugation, single-molecule.

Table of Contents

a) Experimental

S1. Synthesis and characterization....S3

S2. NMR spectra of reported compounds....S9

S3. Crystallographic data....S16

S4. Photophysical measurements....S18

S5. Conductance measurements....S19

S6. Seebeck coefficient measurements....S21

b) Theoretical

S7. Theoretical Methods....S23

S8. Molecular Orbitals....S24

S9. Energy levels, ionization potential and electron affinity....S26

S10. Molecular junction geometries....S27

S10. Seebeck coefficient calculations....S30

S11. Molecule **4** transport calculations....S31

S12. Energy of molecular junctions S32

References....S36

a) Experimental

S1. Synthesis and characterization of target compounds

Instrumentation. NMR spectra were recorded in deuterated solvent solutions on a Varian VNMRS-600 spectrometer and referenced against solvent resonances (^{1}H, ^{13}C). ASAP data were recorded on a Xevo Q-TOF (Waters) high resolution, an accurate mass tandem mass spectrometer equipped with Atmospheric Pressure Gas Chromatography (APGC) and Atmospheric Solids Analysis Probe (ASAP). Microanalyses were performed by the Elemental Microanalysis Service, Durham University, UK on an Exeter Analytical E-440 machine.

General details. All chemicals were sourced from standard chemical suppliers, with the exception of [4-(methylthio)phenyl]acetylene,[1] 4,4'-bis(pinacolatoboron)benzophenone,[2] bis-(4-bromophenyl)methanol,[3] and bis-(4-pinacolatoboron-phenyl)methanol[4] which were prepared following literature procedures.

1a - *1,5-Bis[4-(methylthio)phenyl]penta-1,4-diyn-3-one*

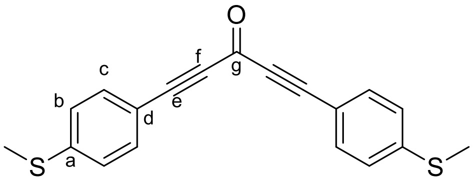

This compound was synthesized following a reported literature procedure.[5] To a CH_2Cl_2 (15 mL) solution of 1,5-bis(4-(methylthio)phenyl)penta-1,4-diyn-3-ol (**5**) (212 mg, 0.653 mmol) was added MnO_2 (114 mg, 1.31 mmol). After stirring at room temperature for 2 h, the suspension was purified by preparative column chromatography on a silica gel column using DCM: hexane (1:1, R_f = 0.1) as the eluent to give the product as a yellow solid. **Yield:** 62% (131 mg, 0.406 mmol). **^{1}H NMR** (599 MHz, $CDCl_3$) δ 7.56 – 7.52 (m, 4H, H^c), 7.24 – 7.20 (m, 4H, H^b), 2.50 (s, 6H, H^{SMe}); **^{13}C NMR** (151 MHz, $CDCl_3$) δ 160.6 (C^g), 144.1 (C^a), 133.6

(C^c), 125.4 (C^b), 115.2 (C^d), 92.0 (C^f), 90.1 (C^e), 14.8 (C^{SMe}); **HRMS** (ES^+) *m/z* 323.0578 $[M+H]^+$, calc. for 323.0564 [$C_{19}H_{15}OS_2$].

2a - *4,4'-(dimethylthio)benzophenone*

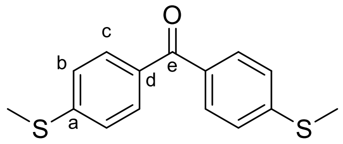

This compound was prepared following the reported literature procedure for compound **1a**.[5]

MnO_2 (213 mg, 2.45 mmol) was added to a solution of **6** (339 mg, 1.23 mmol) in CH_2Cl_2 (20 mL) and stirred at room temperature for 2 h. The residue was purified by column chromatography on silica with DCM: hexane (1:1, R_f = 0.3 in 80:20) as the eluent to give the product as a white solid. **Yield**: 41% (139 mg, 0.507 mmol). **^{1}H NMR** (599 MHz, CD_2Cl_2) δ 7.75 – 7.66 (m, 4H, H^c), 7.35 – 7.27 (m, 4H, H^b), 2.54 (s, 6H, H^{Me}); **^{13}C NMR** (151 MHz, CD_2Cl_2) δ 194.4 (C^e), 144.9 (C^a), 133.9 (C^d), 130.3 (C^c), 124.7 (C^b), 14.6 (C^{Me}); **HRMS** (ES^+) *m/z* 275.0568 $[M+H]^+$, calc. for 275.0564 [$C_{15}H_{15}OS_2$]; **Anal. Calc.** for $C_{15}H_{14}OS_2$: C, 65.66; H, 5.41; N, 0.00 %; **Found:** C, 65.55; H, 5.10; N 0.21 %. The experimental data obtained were in good agreement with the literature, where the compound was synthesized *via* a different route.[6]

3a

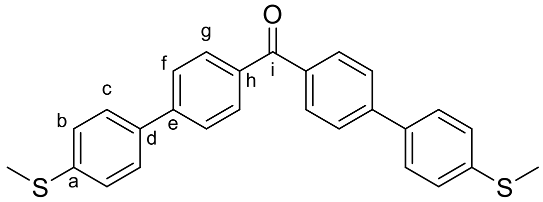

4,4'-Di(bispinacolatoboron)benzophenone (530 mg, 1.22 mmol), 4-bromothioanisole (496 mg, 2.44 mmol) and 1 M aqueous Na_2CO_3 (1040 mg, 9.77 mmol) were added to a Schlenk flask with DME (10 mL) and water (10 mL) and degassed by argon sparging. $Pd(PPh_3)_4$ (71 mg,

0.061 mmol) was added under a flow of argon and the reaction mixture heated to 85 °C for 24 h. The reaction mixture was cooled to RT, followed by the addition of water (20 mL) and extraction of the product with DCM (3 × 20 mL). The combined organics were dried over $MgSO_4$, filtered and the solvent removed under reduced pressure. The residue was purified by column chromatography on silica with hexane: ethyl acetate as the eluent (R_f = 0.5 in 70:30) to give the product as a white solid. **Yield:** 7% (37 mg, 0.087 mmol). **^{1}H NMR** (599 MHz, $CDCl_3$) δ 7.93 – 7.88 (m, 4H, H^g), 7.71 – 7.66 (m, 4H, H^f), 7.61 – 7.55 (m, 4H, H^c), 7.38 – 7.32 (m, 4H, H^b), 2.53 (s, 6H, H^{Me}); **^{13}C NMR** (151 MHz, $CDCl_3$) δ 195.7 (C^i), 144.4 (C^e), 139.0 (C^a), 136.7 (C^d), 136.2 (C^h) 130.7 (C^g), 127.6 (C^c), 126.8 (C^b), 126.6 (C^f), 15.6 (C^{Me}); **HRMS** (ES^+) *m/z* 427.1195 $[M+H]^+$, calc. for 427.1190 [$C_{27}H_{23}OS_2$]; **Anal. Calc.** for $C_{27}H_{22}OS_2·0.1CH_2Cl_2$: C. 74.91; H, 5.14; N, 0.00 %; **Found:** C, 75.18; H, 5.16; N, −0.18 %. 2D NMR experiments (HSQC and HMBC) were used to aid assignments of the ^{13}C spectrum as the compound has very limited solubility in standard NMR solvents.

1b - *1,5-Bis[4-(methylthio)phenyl]penta-1,4-diyn-3-ol*

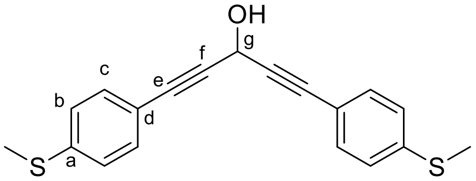

This compound was prepared following a reported literature procedure.[5] A dry hexane (5 mL) solution of [4-(methylthio)phenyl]acetylene (194 mg, 1.31 mmol) was cooled to −30 °C 1.6 M *n*-BuLi (0.743 mL, 0.595 mmol) was then added dropwise. After stirring for 1 h, the white suspension was cooled to −78 °C. Ethyl formate (0.142 mL, 1.76 mmol) was added followed by 30 min stirring. After gradually rising to room temperature, the reaction was stirred for 24 h. Saturated NH_4Cl aqueous solution was added to quench the reaction, followed by extraction of the product with ethyl acetate (3 × 50 mL). The organic phase was combined and dried over $MgSO_4$. After filtration and removal of the solvent, the residue was purified *via* preparative column chromatography on a silica gel column using a hexane: DCM gradient (R_f = 0.3 in 40:60 hexane: DCM) as the eluent to give the product as a pale yellow solid. **Yield:** 50% (288 mg, 0.888 mmol). **^{1}H NMR** (599 MHz, CD_2Cl_2) δ 7.50 – 7.35 (m, 4H, H^c), 7.29 – 7.10 (m, 4H,

H^b^), 5.56 (d, J = 7.3 Hz, 1H, H^g), 2.49 (s, 6H, H^{Me}); **^{13}C NMR** (151 MHz, CD_2Cl_2) δ 140.5 (C^a), 132.0 (C^c), 125.6 (C^b), 117.9 (C^d), 86.0 (C^f), 84.0 (C^e), 53.0 (C^g), 14.9 (C^{Me}); **HRMS** (ES^+) *m/z* 307.0623 $[M\text{-}OH]^+$, calc. for 307.0615 [$C_{19}H_{15}S_2$]; **Anal. Calc.** for $C_{19}H_{16}OS_2{\cdot}0.25CH_2Cl_2$: C, 66.88; H, 4.81; N, 0.00 %; **Found:** C, 66.89; H, 4.66; N −0.02 %.

2b - *Bis(4-(methylthio)phenyl)methanol*

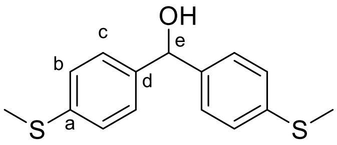

This compound was prepared following a reported literature procedure.[7] 4-Bromothioanisole (1.00 g, 4.92 mmol) was added to an oven-dried flask with dry THF (15 mL), cooled to −78 °C and stirred for 5 mins. 2.5 M *n*-BuLi in hexane (0.347 g, 5.42 mmol) was added slowly and stirred at −78 °C for 90 minutes before addition of neat 4-(methylthio)benzaldehyde (0.937 g, 6.16 mmol). The reaction mixture was stirred at −78 °C for 15 minutes, then slowly warmed to RT and stirred for a further 1 h. The mixture was quenched by the addition of water, and the aqueous layer extracted with ethyl acetate (3 × 10 mL), washed with brine, and the combined organics dried over $MgSO_4$. The residue was purified by column chromatography on silica with hexane: ethyl acetate as the eluent (R_f = 0.3 in 80:20 hexane: ethyl acetate) to give the product as a white solid. **Yield:** 33% (447 mg, 1.62 mmol). **^{1}H NMR** (599 MHz, CD_2Cl_2) δ 7.31 – 7.26 (m, 4H, H^c), 7.23 – 7.20 (m, 4H, H^b), 5.76 (d, J = 1.9 Hz, 1H, H^e), 2.46 (s, 6H, H^{Me}); **^{13}C NMR** (151 MHz, CD_2Cl_2) δ 140.9 (C^d), 137.8 (C^a), 126.9 (C^c), 126.4 (C^b), 75.2 (C^e), 15.5 (C^{Me}); **HRMS** (ES^+) *m/z* 259.0621 $[M\text{-}OH]^+$, calc. for 259.0615 [$C_{15}H_{15}S_2$]; **Anal. Calc.** for $C_{15}H_{16}OS_2$: C, 65.18; H, 5.83; N, 0.00 %; **Found:** C, 64.80; H, 5.78; N 0.21 %.

3b

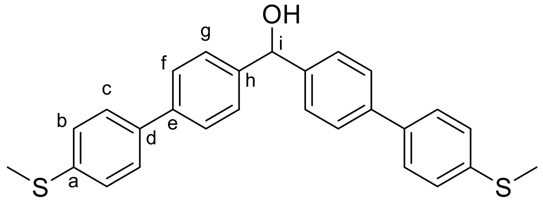

Bis-(4-bispinacolatoboron-phenyl)methanol (424 mg, 0.972 mmol), 4-bromothioanisole (395 mg, 1.94 mmol) and 1 M aqueous Na_2CO_3 (824 mg, 7.78 mmol) were added to a Schlenk flask with DME (8 mL) and water (8 mL) and degassed by argon sparging. $Pd(PPh_3)_4$ (56 mg, 0.049 mmol) was added under a flow of argon and the reaction mixture heated to 85 °C for 24 h. The reaction mixture was cooled to RT, followed by the addition of water (20 mL) and extraction of the product with DCM (3 × 20 mL). The combined organics were dried over $MgSO_4$, filtered and the solvent removed under reduced pressure. The residue was purified by column chromatography on silica with hexane: ethyl acetate as the eluent (R_f = 0.3 in 80:20) to give the product as a white solid. **Yield**: 24% (99 mg, 0.231 mmol). **^{1}H NMR** (599 MHz, $CDCl_3$) δ 7.56 – 7.53 (m, 4H, H^f), 7.51 – 7.48 (m, 4H, H^c), 7.48 – 7.46 (m, 4H, H^g), 7.33 – 7.29 (m, 4H, H^b), 5.92 (d, J = 3.4 Hz, 1H, H^i), 2.51 (s, 6H, H^{Me}), 2.26 (d, J = 3.5 Hz, 1H, H^{OH}); **^{13}C NMR** (151 MHz, $CDCl_3$) δ 142.7 (C^h), 139.9 (C^e), 137.7 (C^a), 137.5 (C^d), 127.4 (C^c), 127.0 (C^f), 126.9 (C^g), 126.9 (C^b), 75.9 (C^i), 15.9 (C^{Me}); **HRMS** (ES^+) *m/z* 411.1227 $[M-OH]^+$, calc. for 411.1241 $[C_{27}H_{23}S_2]$; **Anal. Calc.** for $C_{27}H_{24}OS_2 \cdot 0.2C_4H_8O_2$: C, 74.83; H, 5.78; N, 0.00 %; **Found:** C, 74.75; H, 6.16; N −0.06 %.

4

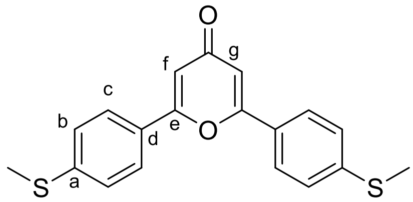

This compound was prepared following a reported literature procedure for analogs.[8] To a solution of 1,5-bis[4-(methylthio)phenyl]penta-1,4-diyn-3-one (**1**) (32 mg, 0.10 mmol) in

methanol (1 mL) was added 15 mol% of *p*-toluenesulfonic acid (*p*-TsOH) (3 mg, 0.015 mmol). The resulting mixture was stirred at 110 °C in a sealed tube. When the reaction was considered complete, determined by TLC (approx. 5 h), the reaction mixture was diluted with diethyl ether (30 mL), washed with water and saturated brine, dried over $MgSO_4$, and the solvent removed under reduced pressure. The residue was purified by column chromatography on silica using hexane: ethyl acetate as the eluent (R_f = 0.3 in ethyl acetate) to give the product as a pale yellow solid. Crystals for X-ray analysis were grown by cooling a solution of the compound in DCM/hexane. **Yield**: 15% (5 mg, 0.015 mmol). **^{1}H NMR (**599 MHz, $CDCl_3$) δ 7.76 – 7.72 (4 H, m, H^c), 7.37 – 7.31 (4 H, m, H^b), 6.75 (2 H, s, H^f), 2.54 (6 H, s, H^{Me}); **^{13}C NMR** (151 MHz, $CDCl_3$) δ 180.2 (C^g), 162.9 (C^d), 143.7 (C^a), 127.6 (C^e), 126.1 (C^b), 126.0 (C^c), 110.6 (C^f), 15.0 (C^{Me}); **HRMS** (ES^+) *m/z* 341.0678 $[M+H]^+$, calc. for 341.0670 [$C_{19}H_{17}O_2S_2$]; **Anal. Calc.** for $C_{19}H_{16}O_2S_2$·0.1CH_2Cl_2:[i] C, 65.74; H, 4.68; N, 0.00; **Found**: C, 65.65; H, 4.70; N, 0.03.

[i] The source of the dichloromethane is the solvent used to transfer the compound to the vial for weighing and obtaining the elemental analysis data.

S2. NMR spectra of reported compounds

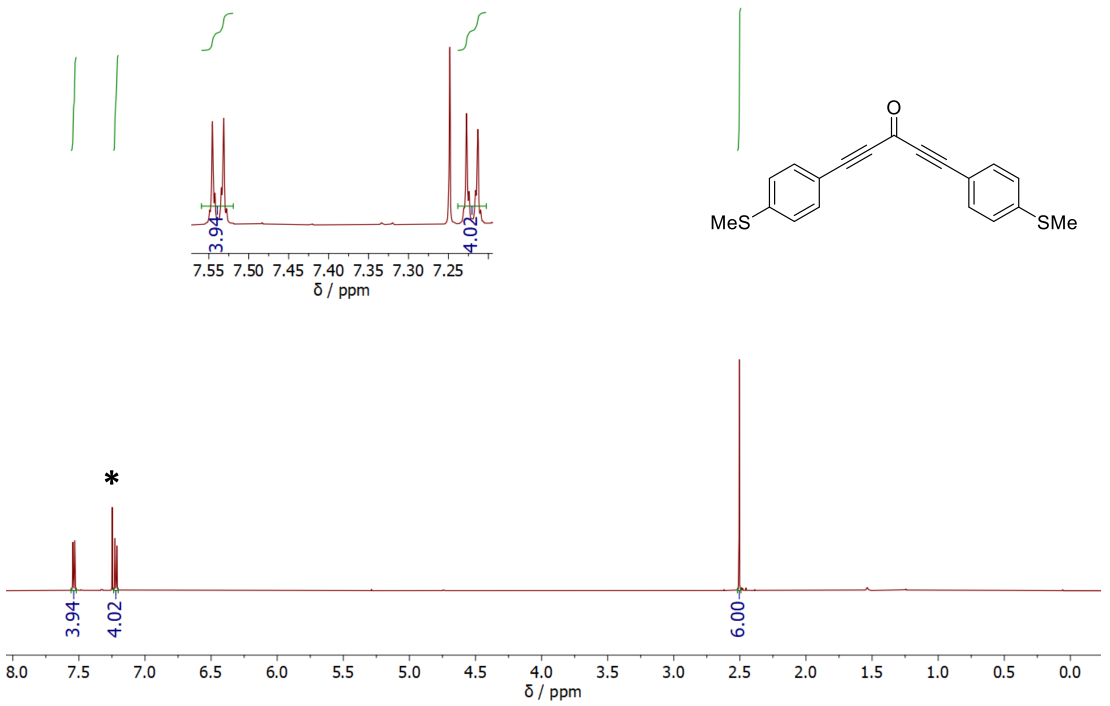

Figure S1. ^{1}H NMR spectrum of compound **1a** recorded in $CDCl_3$ (*residual protio solvent).

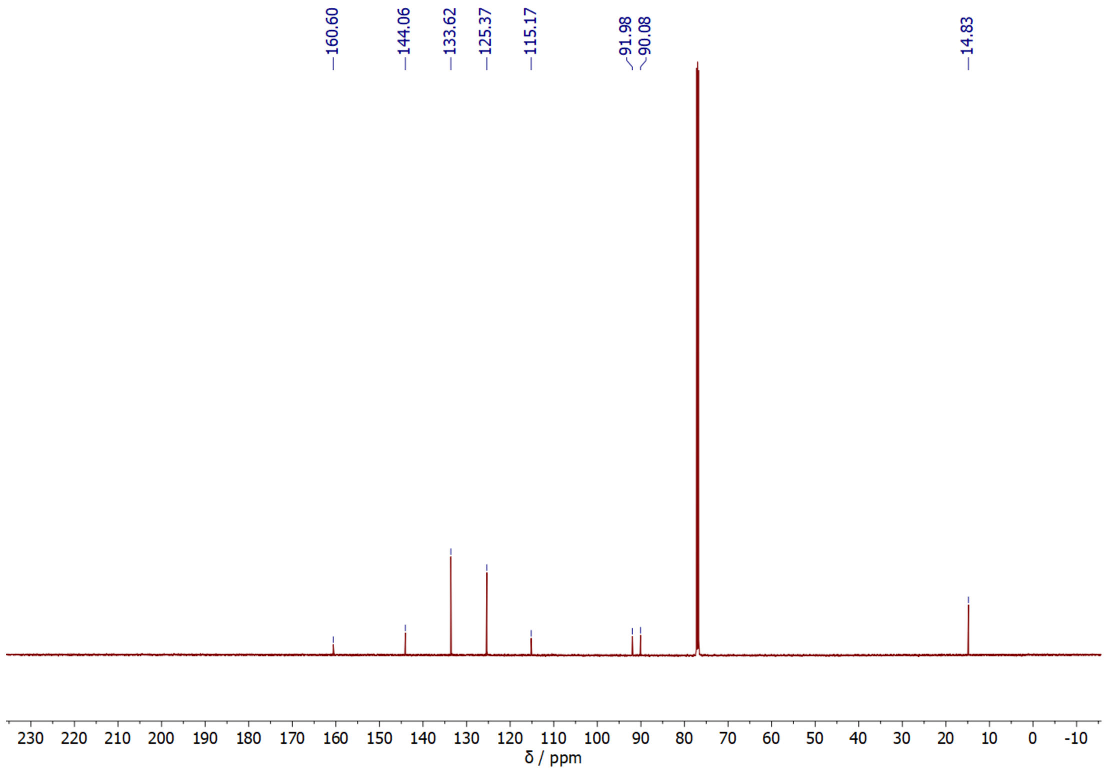

Figure S2. ^{13}C NMR spectrum of compound **1a** recorded in $CDCl_3$.

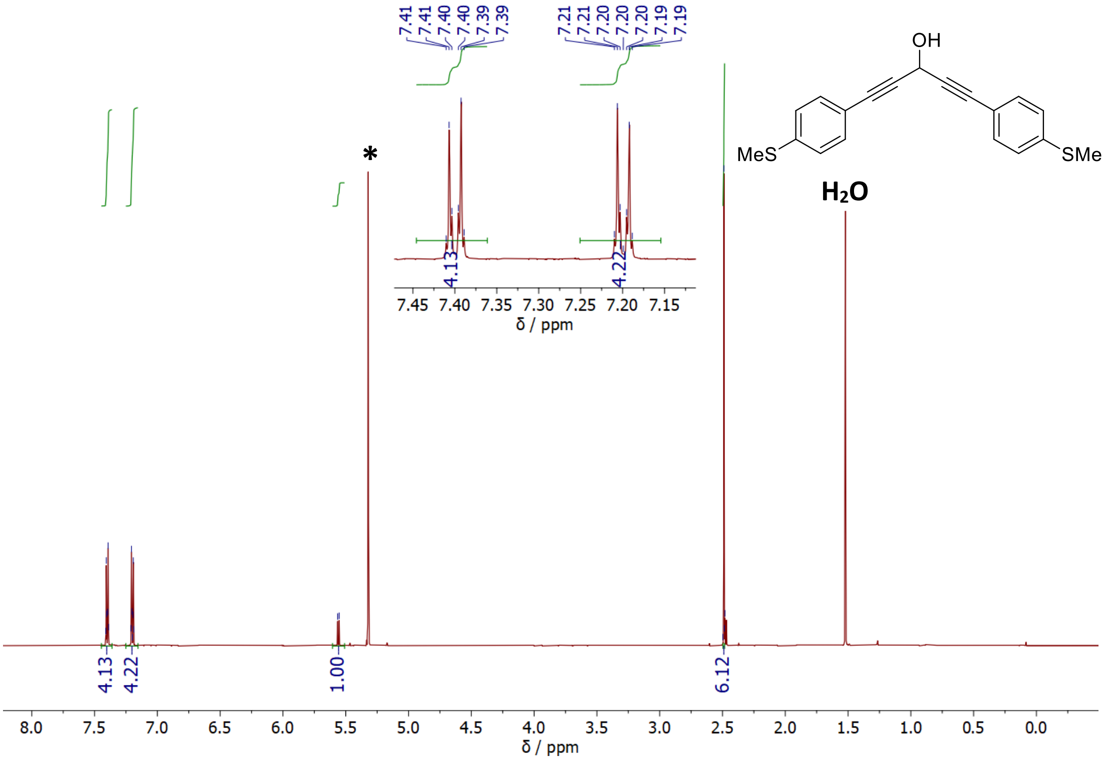

Figure S3. ^{1}H NMR spectrum of compound **1b** recorded in CD_2Cl_2 (*residual protio solvent).

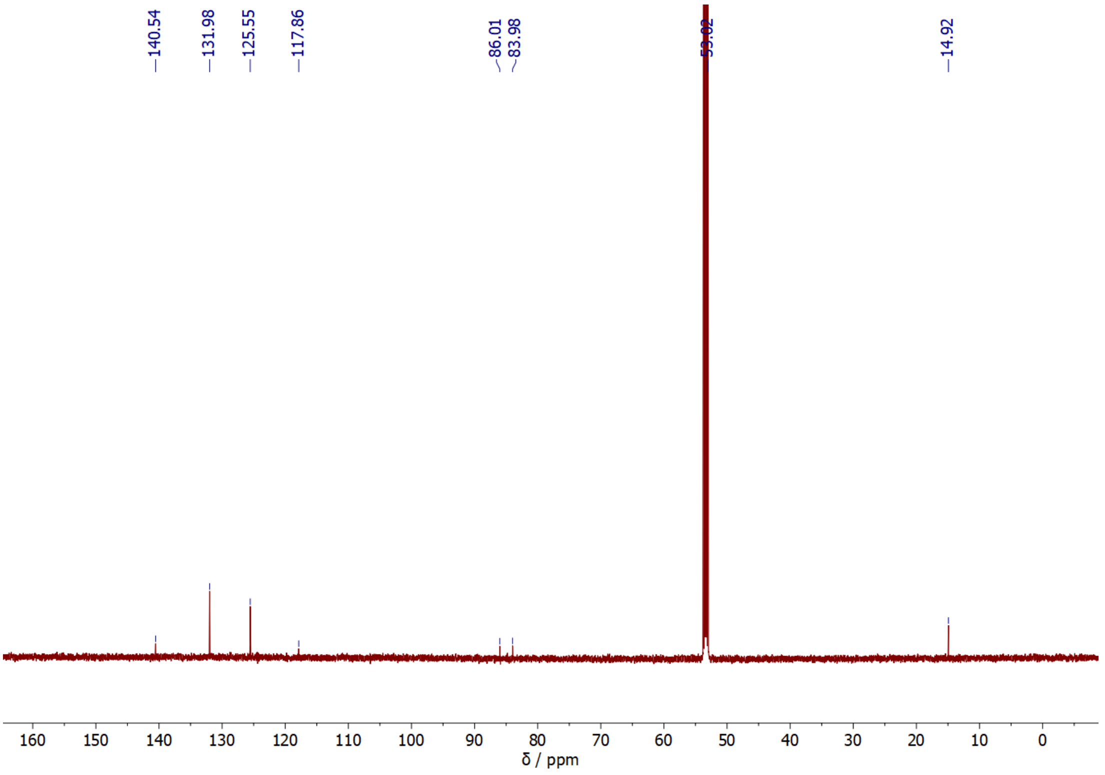

Figure S4. ^{13}C NMR spectrum of compound **1b** recorded in CD_2Cl_2.

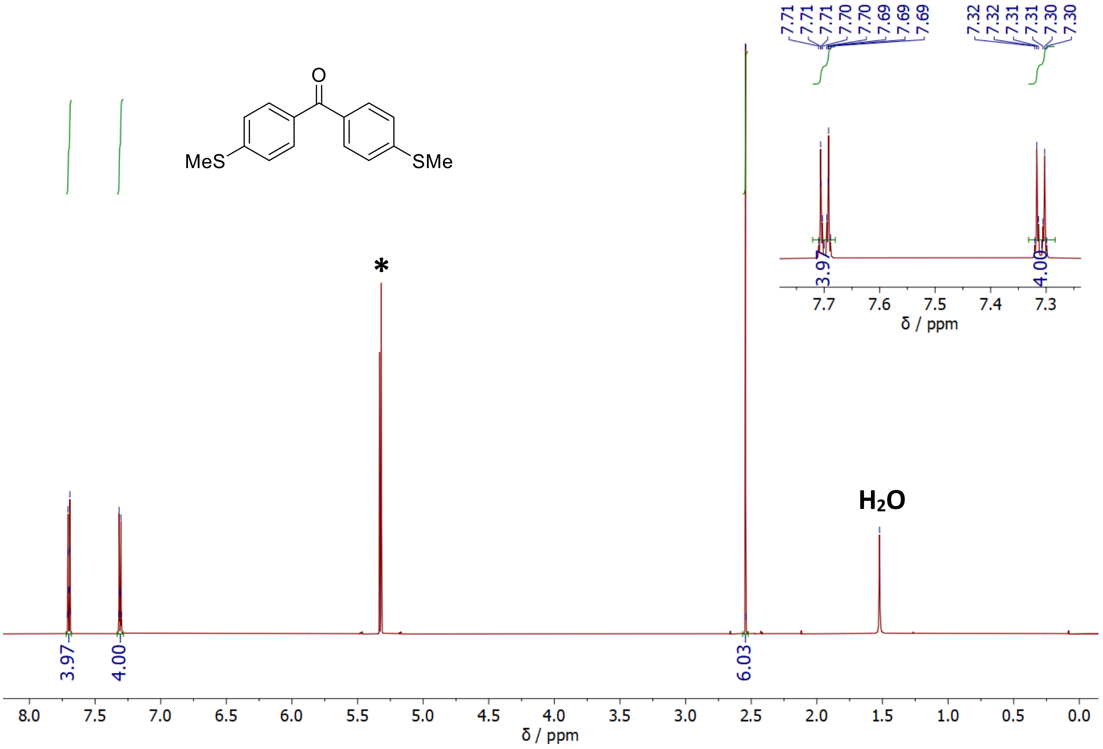

Figure S5. ^{1}H NMR spectrum of compound **2a** recorded in CD_2Cl_2 (*residual protio solvent).

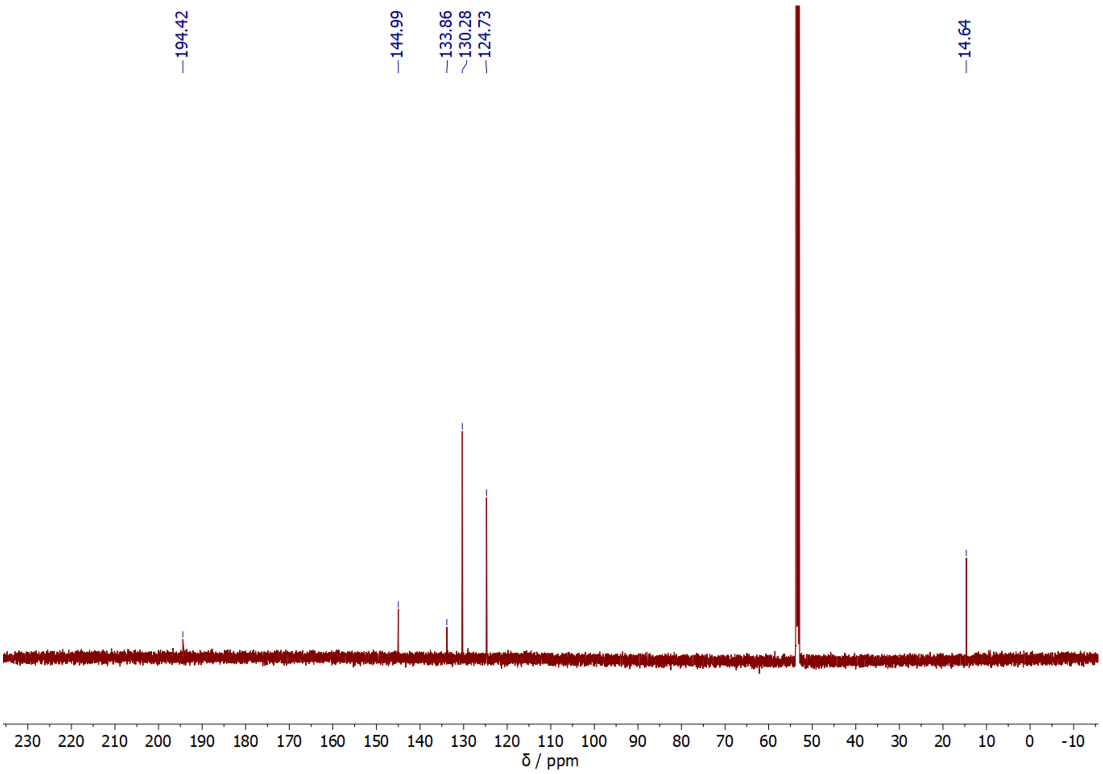

Figure S6. ^{13}C NMR spectrum of compound **2a** recorded in CD_2Cl_2.

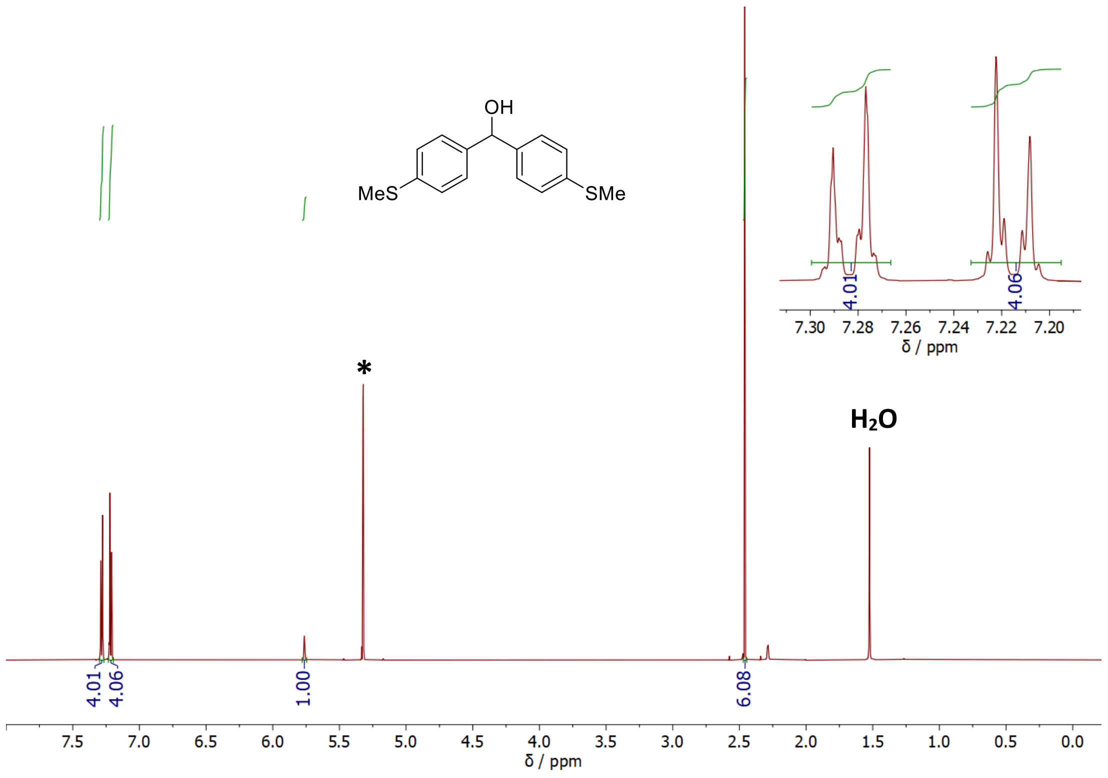

Figure S7. ^{1}H NMR spectrum of **2b** recorded in CD_2Cl_2 (*residual protio solvent).

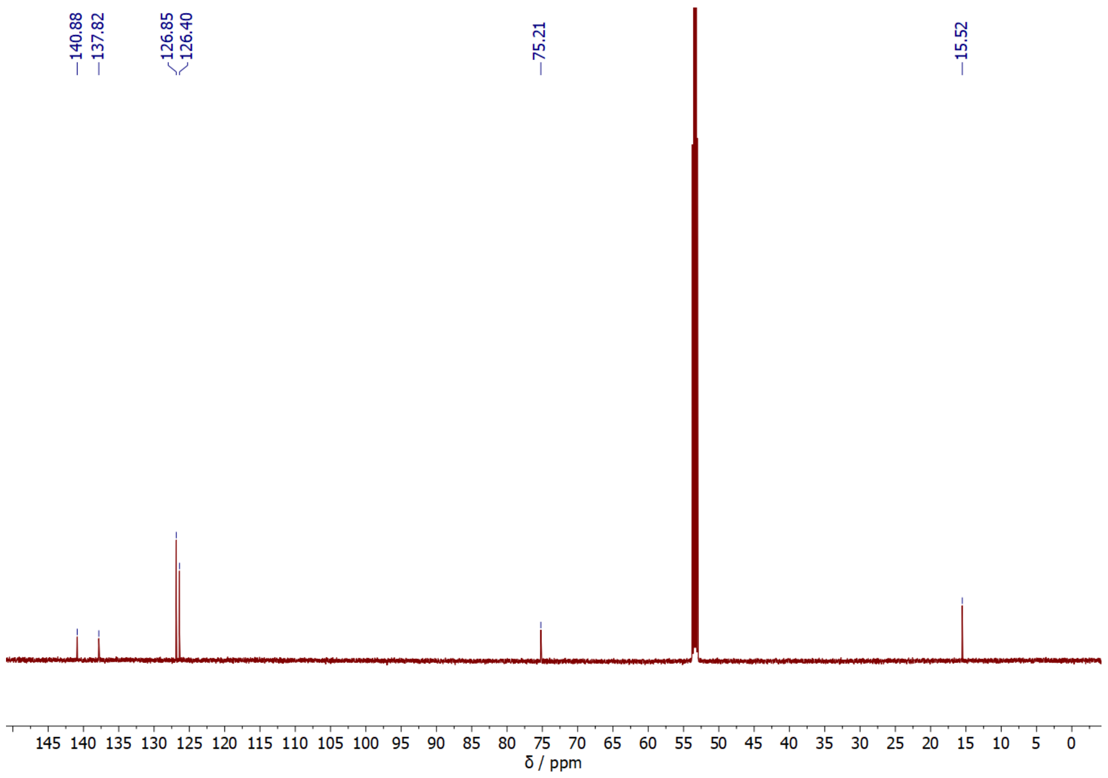

Figure S8. ^{13}C NMR spectrum of compound **2b** recorded in CD_2Cl_2.

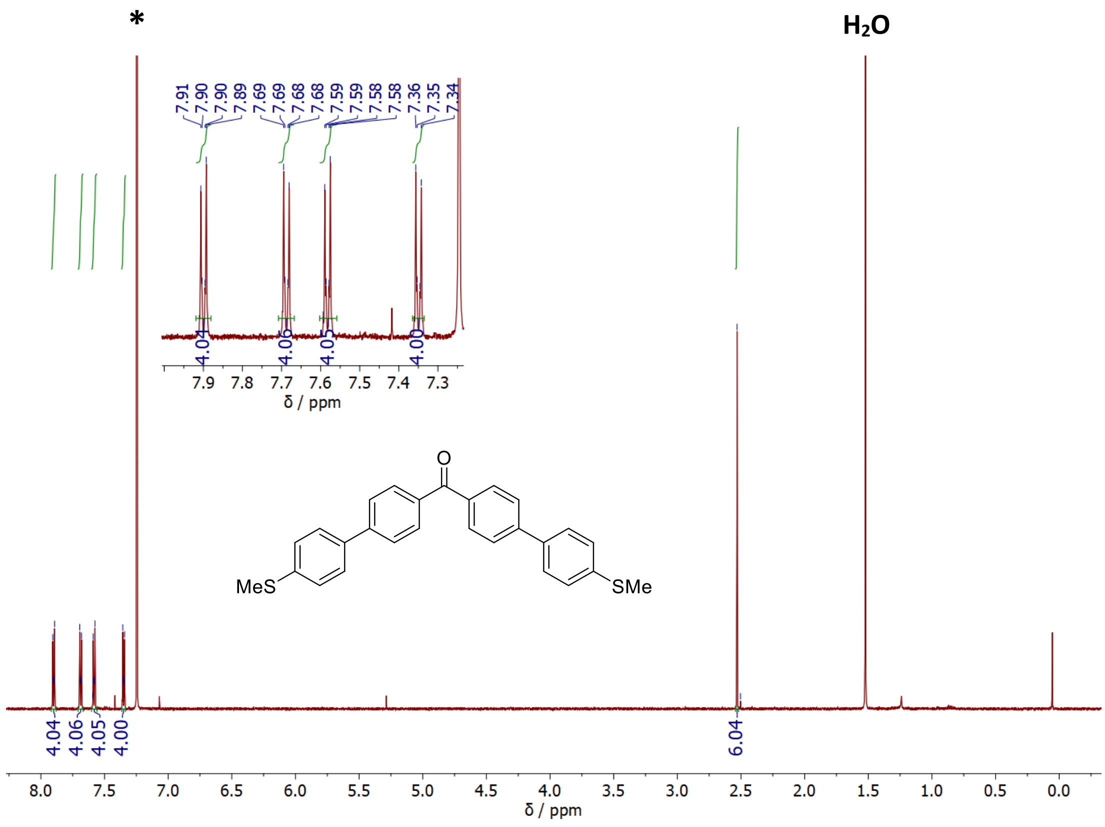

Figure S9. ^{1}H NMR spectrum of **3a** recorded in $CDCl_3$ (*residual protio solvent).

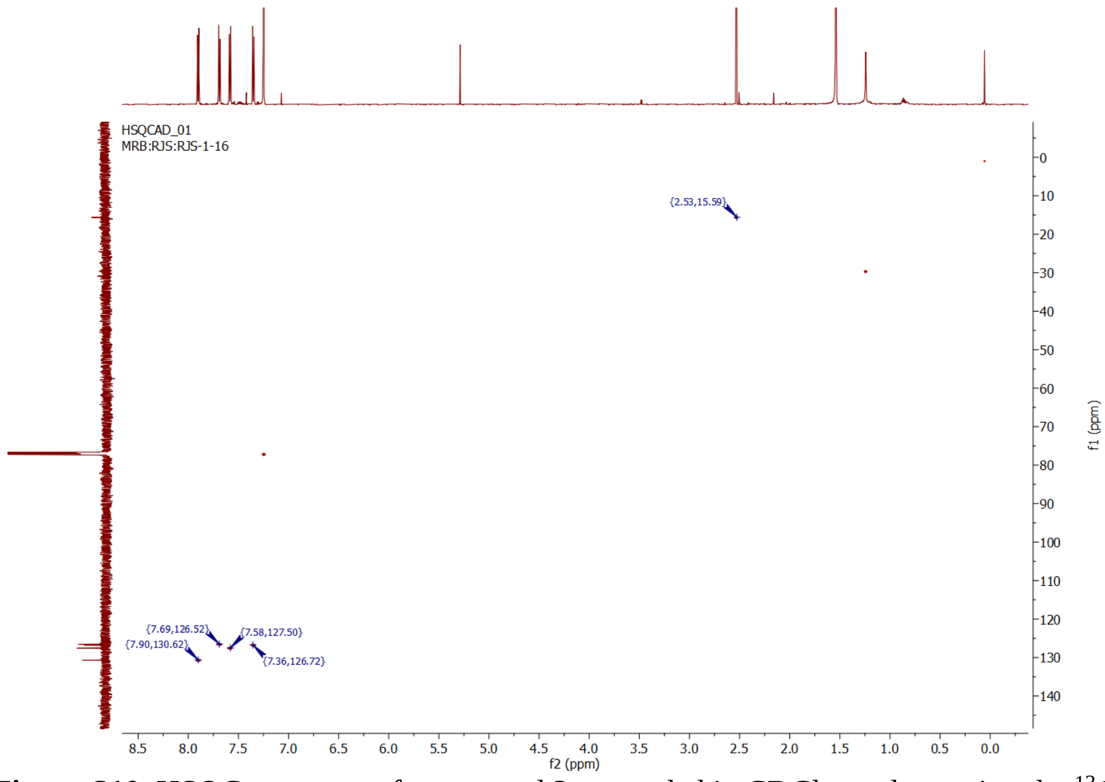

Figure S10. HSQC spectrum of compound **3a** recorded in $CDCl_3$ used to assign the ^{13}C peaks due to the very low solubility of the compound.

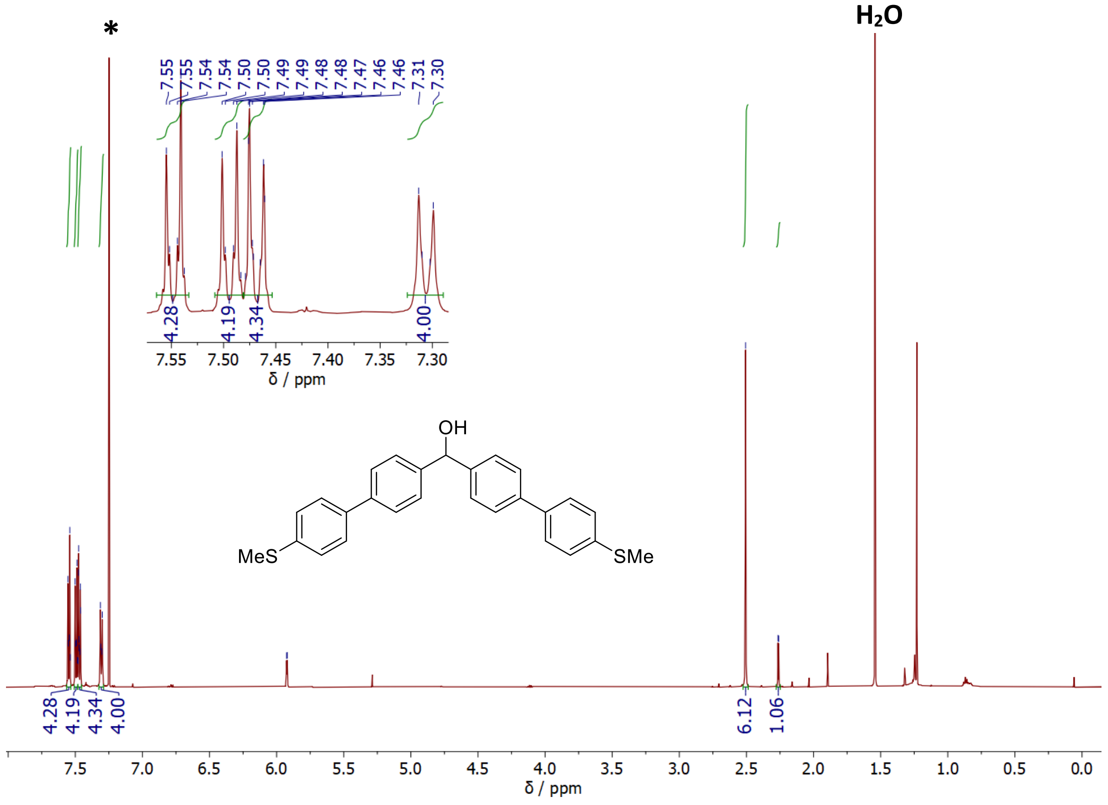

Figure S11. ^{1}H NMR spectrum of **3b** recorded in $CDCl_3$ (*residual protio solvent).

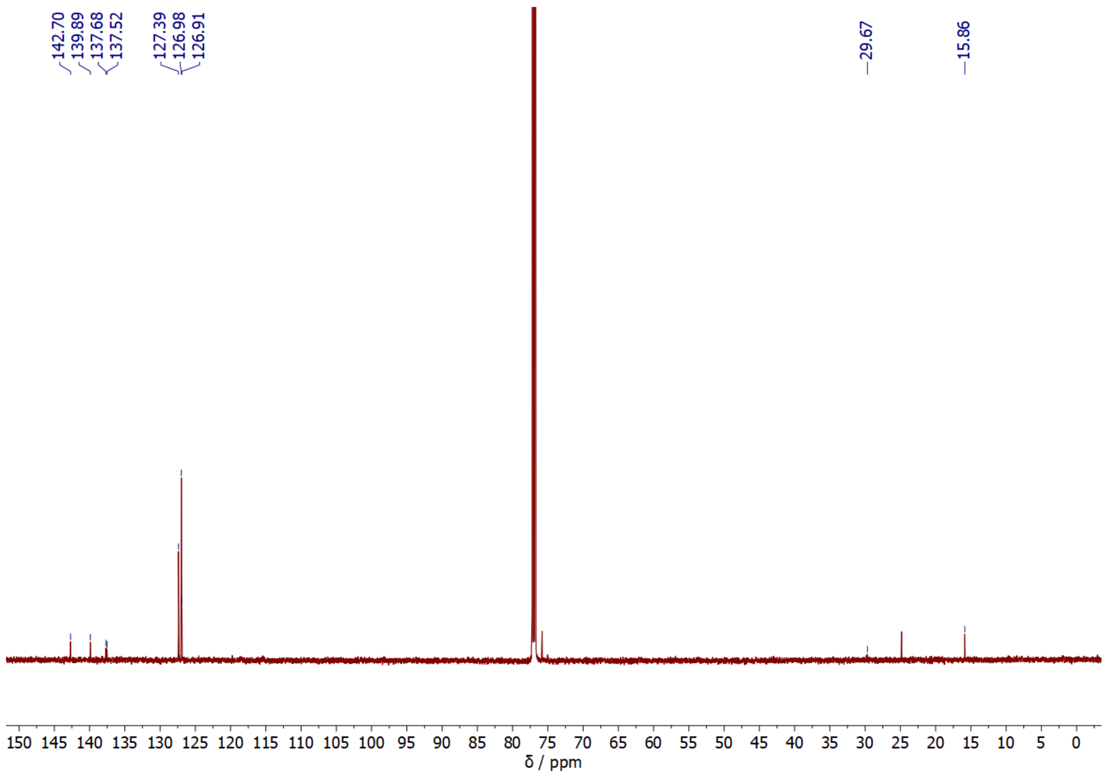

Figure S12. ^{13}C NMR spectrum of compound **3b** recorded in $CDCl_3$.

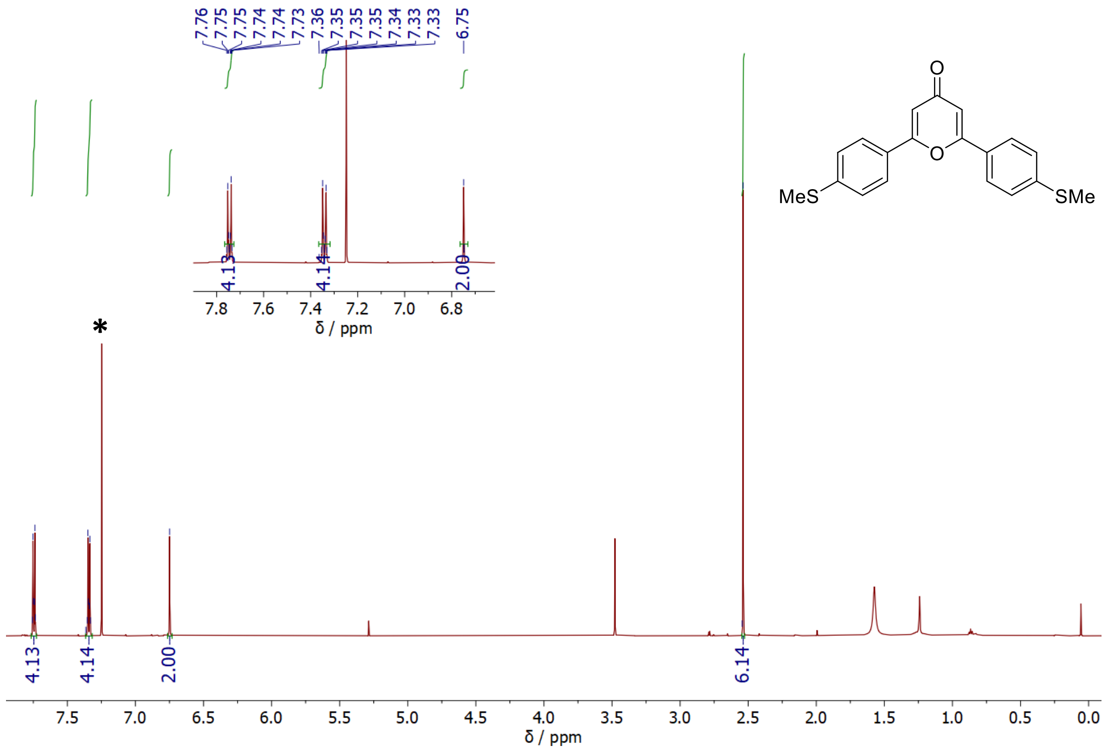

Figure S13. ^{1}H NMR spectrum of compound **4** recorded in $CDCl_3$ (*residual protio solvent).

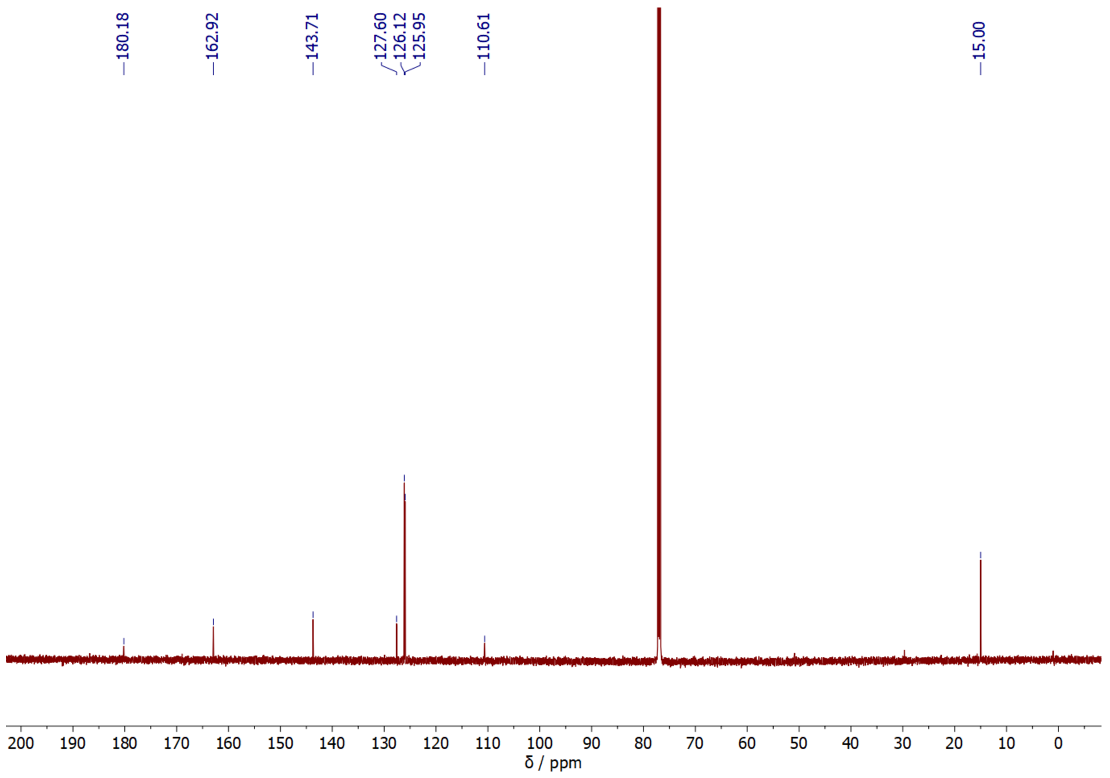

Figure S14. ^{13}C NMR spectrum of compound **4** recorded in $CDCl_3$.

S3. Crystallographic data

Crystals of compound **4** suitable for X-ray diffraction were grown from a solution of the compound in DCM and hexane at 4 °C. The X-ray diffraction experiment was carried out on a Bruker D8 Venture 3-circle diffractometer, equipped with PHOTON III C14 MM CPAD area detector, using Mo-*K*α radiation (λ=0.71073 Å) from Incoatec IμS 3.00 microsource with focusing mirrors. The temperature 120.0(2)K was maintained by Cryostream 700 (Oxford Cryosystems) open-flow N_2 gas cryostat. The data were collected in shutterless mode by narrow (1°) frame ω-scans covering full sphere of reciprocal space, using APEX3 v. 2019.1-0 software, reflection intensities integrated using SAINT v. 8.40A software (Bruker AXS, 2019). Data were corrected for absorption by semi-empirical method based on Laue equivalents and multiple scans, using SADABS v. 2016/2 software.[9] The structure was solved by direct method using XS program and refined by full-matrix least squares using SHELXL 2018/3 software[10] on OLEX2 platform.[11] Hydrogen atoms were located in the Fourier maps and refined in isotropic mode. Full crystallographic information (including structure factors) has been deposited with Cambridge Crystallographic Data Centre, CSD-2239000.

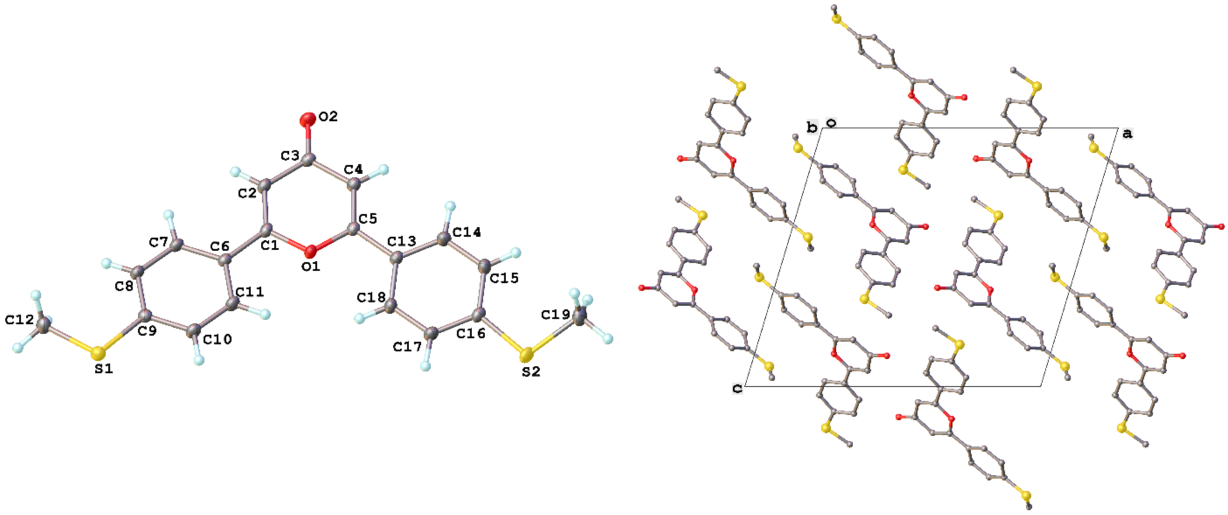

Figure S15. Molecular structure and crystal packing of compound **4**.

Table S1. Crystal data and structure refinement for compound **4**.

Empirical formula	$C_{19}H_{16}O_2S_2$
Formula weight	340.44
Temperature/K	120.00
Crystal system	monoclinic
Space group	$P2_1/c$
a/Å	18.1009(5)
b/Å	5.5511(2)
c/Å	16.5141(5)
α/°	90
β/°	106.6948(11)
γ/°	90
Volume/Å^3	1589.39(9)
Z	4
ρ_{calc}g/cm^3	1.423
μ/mm^{-1}	0.342
F(000)	712.0
Crystal size/mm^3	0.27 × 0.14 × 0.06
Radiation	Mo Kα (λ = 0.71073)
2Θ range for data collection/°	4.698 to 59.992
Index ranges	$-25 \leq h \leq 25$, $-7 \leq k \leq 7$, $-23 \leq l \leq 23$
Reflections collected	41634
Independent reflections	4625 [R_{int} = 0.0520, R_{sigma} = 0.0289]
Data/restraints/parameters	4625/0/272
Goodness-of-fit on F^2	1.084
Final R indexes [I>=2σ (I)]	R_1 = 0.0407, wR_2 = 0.0897
Final R indexes [all data]	R_1 = 0.0500, wR_2 = 0.0935
Largest diff. peak/hole / e Å^{-3}	0.44/-0.28

S4. Photophysical measurements

UV-Visible absorption spectra were recorded at room-temperature using a UV-Visible spectrophotometer Evolution 220 from Thermo Scientific in quartz cuvettes with path length *l* = 1 cm.

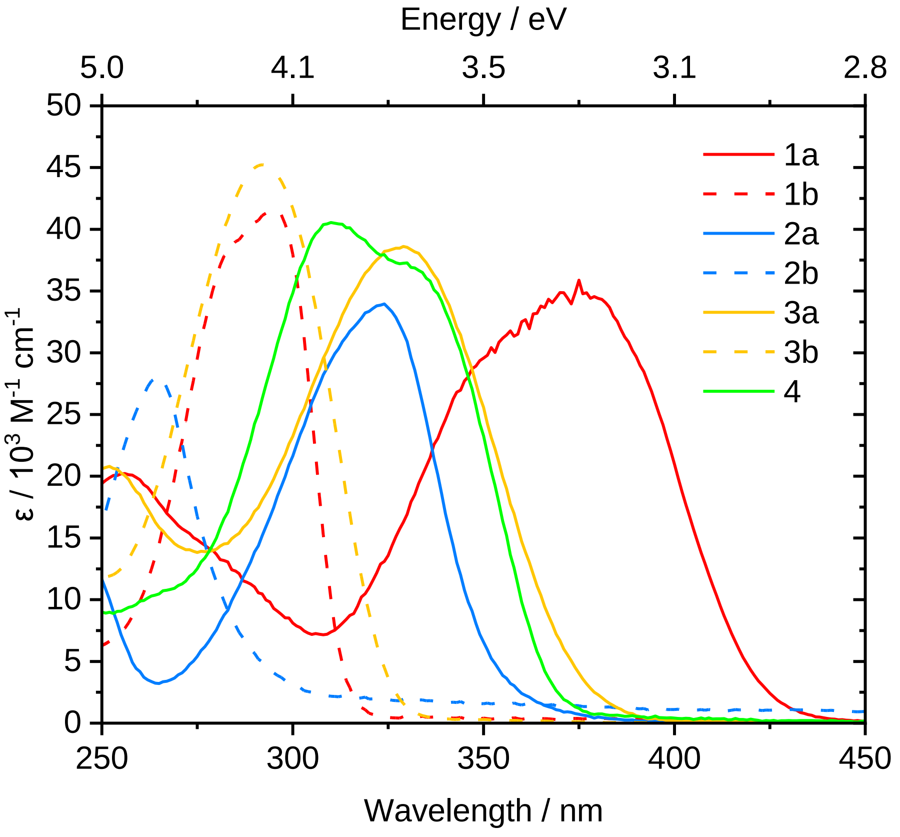

Figure S16. UV-Visible absorption spectra for compounds **1-4** recorded in CH_2Cl_2 (ketone compounds shown as solid lines and their respective alcohol derivatives as dashed lines). As expected, the conjugation to the ketone leads to a red shift in the absorption for these cross-conjugated molecules compared to the alcohol analogs.

S5. Conductance measurements

All compounds were deposited onto Au(111) samples using the drop casting technique. Au samples were annealed at approximately 900 K for 1-2 minutes, allowed to cool down to room temperature and then introduced into a 1 mM dichloromethane (DCM) solution of the corresponding molecule. After 40 minutes, samples were dried off with nitrogen gas to eliminate possible molecular clusters on the surface. Mechanically cut Au wires (0.25 mm diameter, 99.99% purity, Goodfellow) were used as STM tips. A bias voltage was applied to the sample, using $V_{bias} = 100$ mV. The tunnelling current was amplified using a double-stage, home-made, linear current-voltage (I-V)-converter with an overall gain of 2.5 x 10^{10} V/A (5 x 10^{8} V/A in the first stage and multiplied by a factor of 50 in the second one).

The clustering analysis applied was based on the k-means algorithm supported by Matlab. To transform the IZ traces into valid inputs for the algorithm, we assigned a Lorentzian distribution to each conductance point and then summed them all. The number of clusters was initially chosen to be two, which was then successively increased until the complete conductance distribution was properly fitted, without major overlapping between the conductance clusters. With this technique, traces with or without a molecular plateau were separated. Figure S17 shows 2D conductance vs distance histograms of compounds **1a-3a** and **1b-3b**, respectively**.**

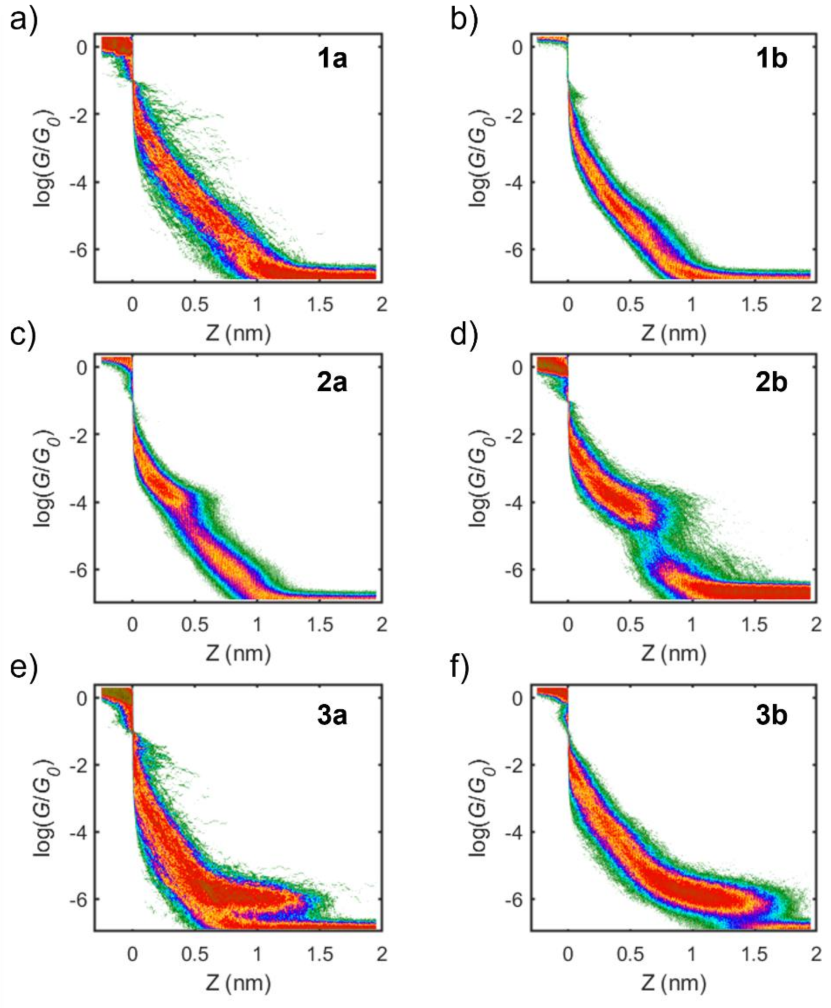

Figure S17. 2D conductance vs distance histograms of compounds **1a-3a** and **1b-3b**.

The apparent stretching length (L_s) of the molecular plateaus were obtained by fitting a Gaussian distribution to each conductance peak and obtaining the length differences between $\overline{G_{1,2}} \pm \sigma_{1,2}$, where $\overline{G_{1,2}}$ and $\sigma_{1,2}$ are the mean conductance value and the standard deviation of the Gaussian fitting curves, respectively. In order to take into account the multiple junction configurations, the Gaussian distributions were fitted to all the distances obtained and the 90% decay distance of the Gaussian fit was then calculated.[12]

The conductance of compound **4** was also measured and is shown in Figure S18. No signature conductance plateau was found for this compound as shown in the 2D *G* vs *z* histogram and 1D *G* histogram.

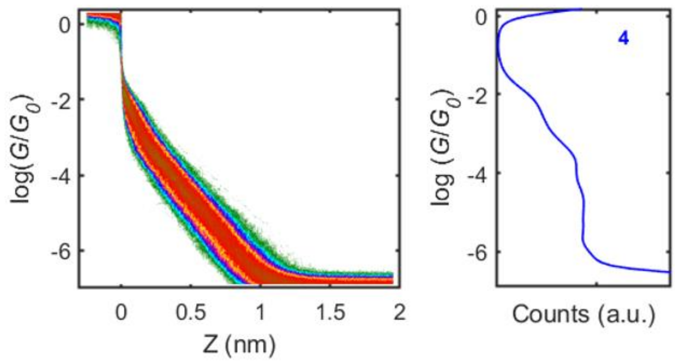

Figure S18. (Left) 2D conductance vs distance histogram and (right) 1D conductance histogram of compound **4**.

S6. Seebeck coefficient measurements

To perform Seebeck coefficient measurements a home-built STM was used, capable of measuring simultaneously the conductance (*G*) and the thermovoltage (V_{th}) of the molecular junctions formed. The tip was heated using a 1 kΩ surface resistor, creating a temperature difference (ΔT) between the tip and the sample, with the tip at $T_h > T_{ambient}$ and the sample at $T_c = T_{ambient}$. This temperature difference not only generates a V_{th} in the molecular junction but also in the copper lead that connects the tip to the rest of the setup. Considering all these factors the thermo-electric equation of the circuit can be expressed as:

$$I = G(V_{bias} + V_{th}) = G(V_{bias} + S\Delta T - S_{lead}\Delta T), \quad (1)$$

where S and S_{lead} are the Seebeck coefficients of the molecule and the copper lead, respectively. Figure S19 shows a scheme of the equivalent electrical-thermal circuit of the STM.

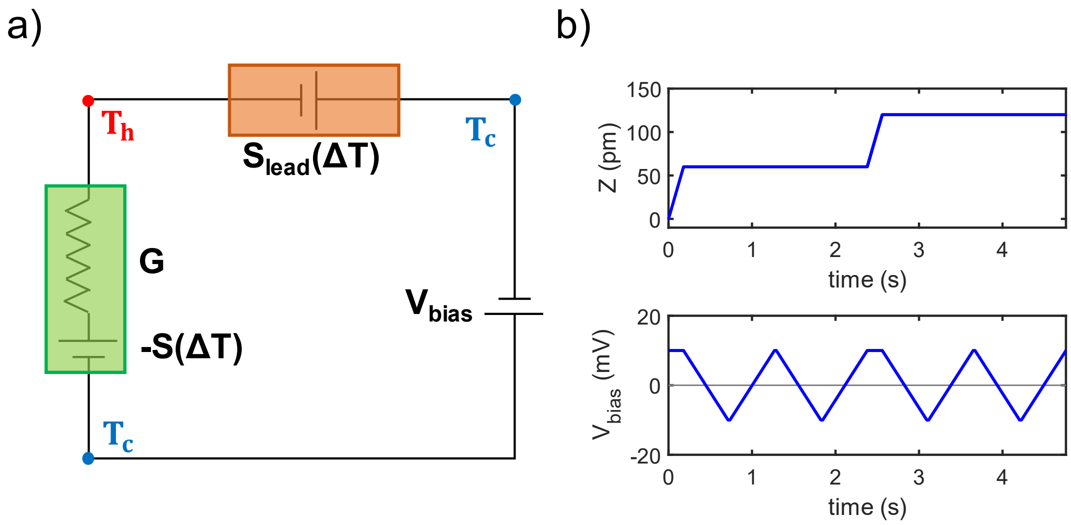

Figure S19. (a) Scheme of the electrical-thermal circuit of the STM, where V_{bias} is the bias voltage applied; S and S_{lead} are the Seebeck coefficients of the molecule and the copper lead, respectively; G is the conductance of the molecular junction, and ΔT is the temperature difference between the tip (at $T_h > T_c$) and the sample ($T_c = T_{ambient}$); (b-c) Tip displacement Z and V_{bias} signals, respectively, during a thermovoltage measurement. While the molecular junction is formed, the tip displacement is momentarily stopped and the V_{bias} is ramped between ±10 mV.

In order to achieve a better stability of the junction, the V_{bias} was fixed at 10 mV, avoiding large voltage changes. While forming the molecular junctions, small IV curves of ±10 mV are acquired to perform the thermoelectric characterization. An example of the tip displacement Z

and the bias voltage V_{bias} signals applied in this case are shown in Figure S18, respectively. For compound **3a**, the V_{bias} was fixed to 40 mV and an amplitude of ± 40 mV for the IV curves. The tip displacement is momentarily stopped during the junction formation and the small IV curves are measured. Applying equation (1), V_{th} and G are simultaneously obtained from the zero-current crossing point and the slope of the IV curves, respectively, and the Seebeck coefficient is then given by $S = -V_{th}/\Delta T$. Multiple sets of V_{th} data were measured for different ΔT values at different days and combined all together in order to obtain more statistically robust results. For consistency, applying the above-mentioned clustering technique we separate the measured V_{th} values into clusters C_1 and C_2, based on the IZ traces. The Seebeck coefficient of each cluster is then obtained from the slope of the linear regression of all V_{th} vs ΔT points.

S7. Theoretical Methods

The optimum geometry of each of the molecules was calculated using the density functional code SIESTA.[13] These used a double-zeta polarized (DZP) basis set defined by a confining cut-off of 0.008 Rydbergs, norm conserving pseudopotentials, an energy cut-off of 150 Rydbergs and the generalized gradient approximation (GGA) method to describe the exchange correlation functional. All forces on the atoms were relaxed to a force tolerance of 0.01 eV/Å. The molecule was then contacted to gold electrodes to form the molecular junction. The gold electrodes were modelled as 6 layers of (111) gold each containing 54 atoms and the SMe anchor groups contact a surface adatom with the optimum Au-S distance calculated to be 2.4Å. A single-zeta basis was used to describe the gold atoms and a Hamiltonian describing this extended molecule was extracted using SIESTA. The zero bias transmission coefficient $T(E)$,

conductance *G* and the Seebeck coefficient, *S*, were calculated using the quantum transport code GOLLUM.[14]

S8. Molecular Orbitals

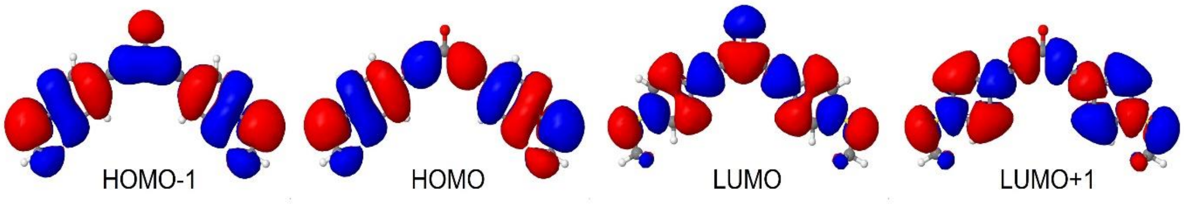

Figure S20. HOMO-1, HOMO, LUMO and LUMO+1 orbitals of molecule **1a**.

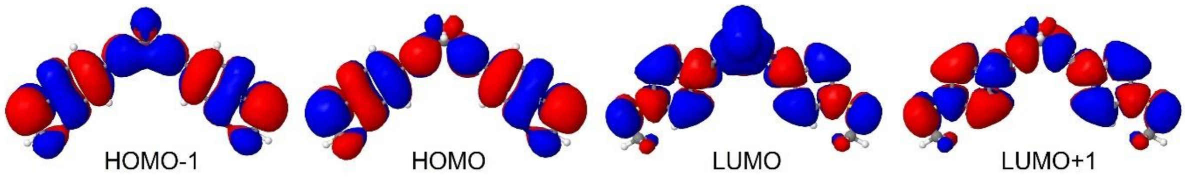

Figure S21. HOMO-1, HOMO, LUMO and LUMO+1 orbitals of molecule **1b**.

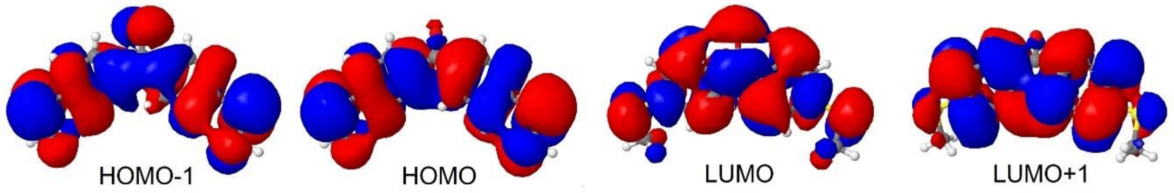

Figure S22. HOMO-1, HOMO, LUMO and LUMO+1 orbitals of molecule **2a**.

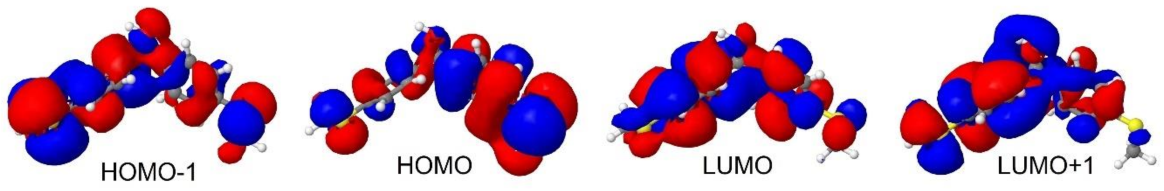

Figure S23. HOMO-1, HOMO, LUMO and LUMO+1 orbitals of molecule **2b**.

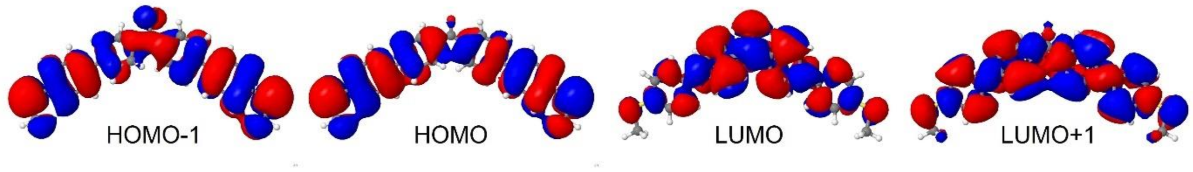

Figure S24. HOMO-1, HOMO, LUMO and LUMO+1 orbitals of molecule **3a**.

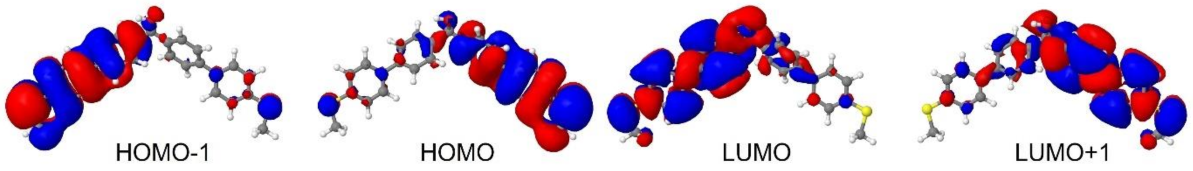

Figure S25. HOMO-1, HOMO, LUMO and LUMO+1 orbitals of molecule **3b**.

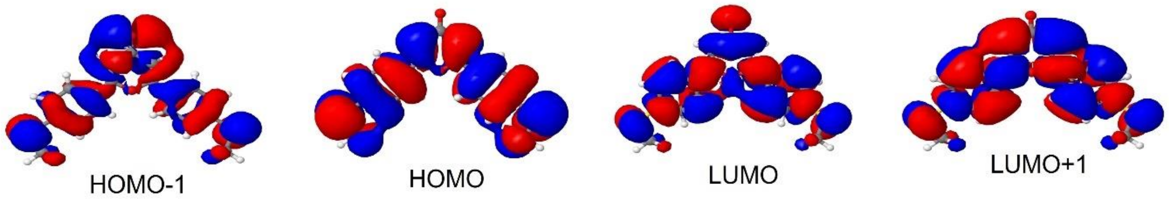

Figure S26. HOMO-1, HOMO, LUMO and LUMO+1 orbitals of molecule **4**.

S9. Energy levels, ionization potential and electron affinity

Table S2. Frontier energy levels for molecules **1-4**.

	HOMO-1 (eV)	HOMO (eV)	LUMO (eV)	LUMO+1(eV)
**1a**	-4.77	-4.62	-2.51	-1.32
**1b**	-4.42	-4.35	-1.36	-1.05
**2a**	-4.64	-4.59	-1.98	-0.87
**2b**	-4.42	-4.14	-0.68	-0.65
**3a**	-4.44	-4.43	-2.22	-1.30
**3b**	-4.37	-4.20	-1.28	-0.94
**4**	-4.61	-4.59	-1.89	-1.66

Table S3. Ionization potential (IP) and electron affinity (EA) for molecules **1-4**.

	IP (eV)	EA (eV)
**1a**	6.31	0.61
**1b**	6.05	-0.37
**2a**	6.56	-0.18
**2b**	6.17	-1.42
**3a**	5.99	0.41
**3b**	5.82	-0.40
**4**	6.33	0.01

S10. Molecular junction geometries

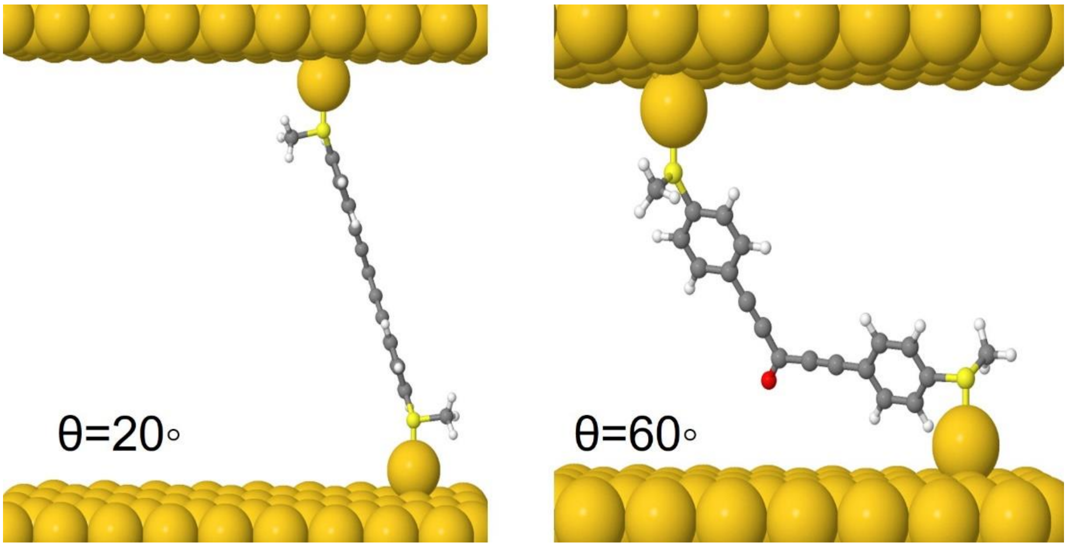

Figure S27. Junction geometry for molecule **1a** connected to gold electrodes *via* a gold adatom for a tilt angle of 20° (left) and 60° (right).

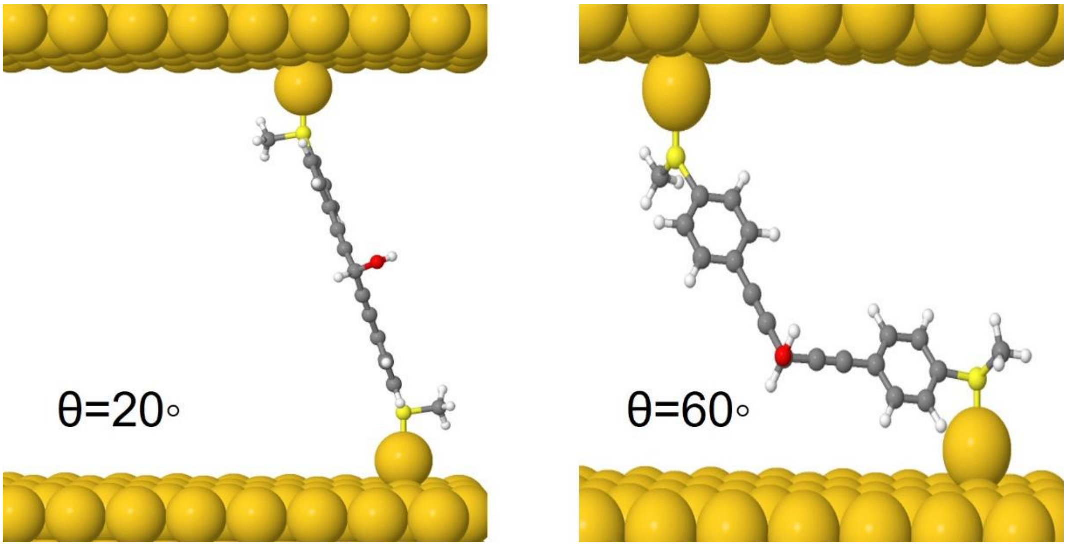

Figure S28. Junction geometry for molecule **1b** connected to gold electrodes *via* a gold adatom for a tilt angle of 20° (left) and 60° (right).

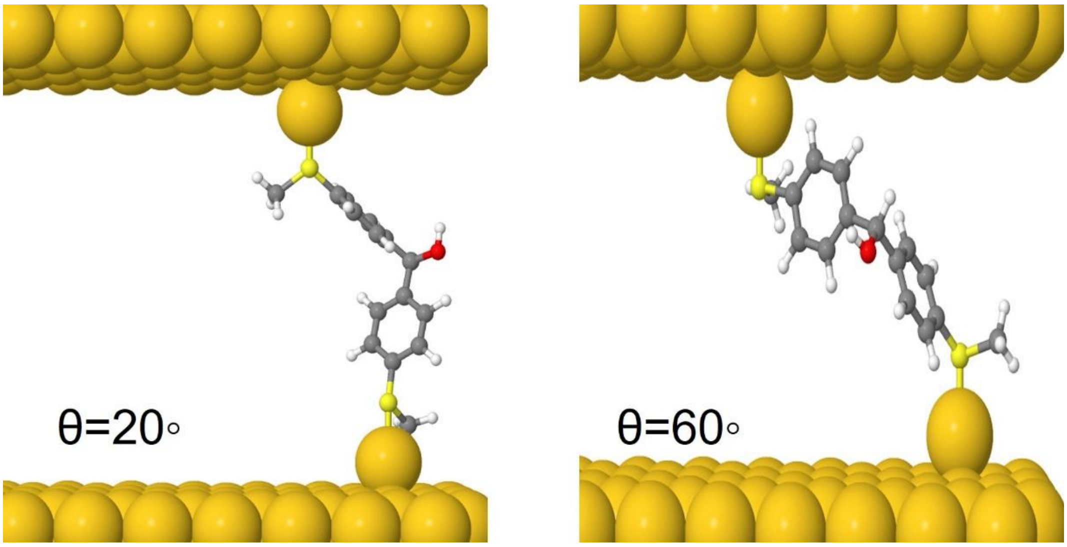

Figure S29. Junction geometry for molecule **2b** connected to gold electrodes *via* a gold adatom for a tilt angle of 20° (left) and 60° (right).

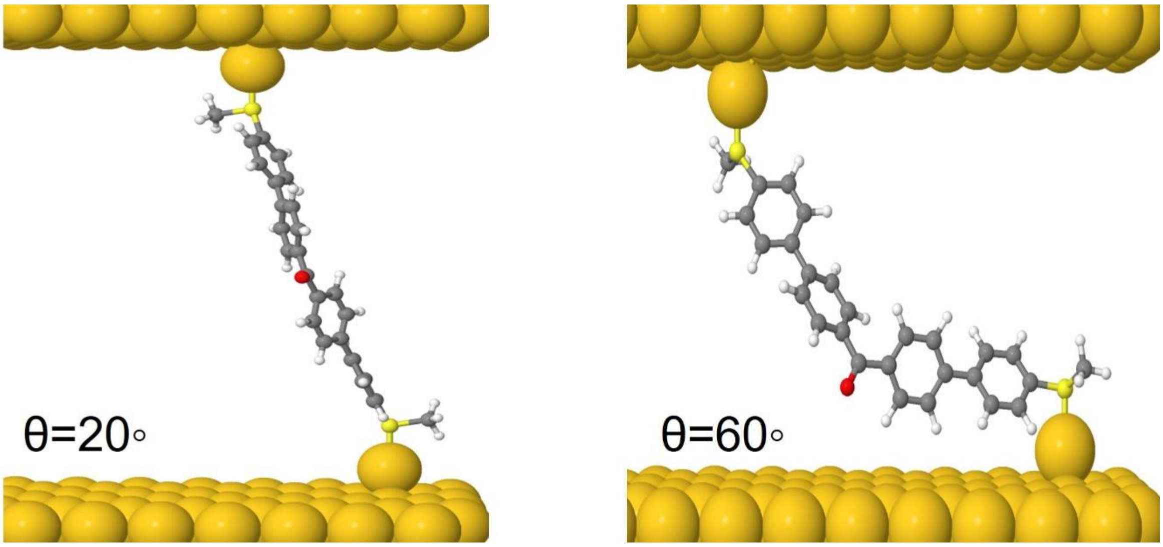

Figure S30. Junction geometry for molecule **3a** connected to gold electrodes *via* a gold adatom for a tilt angle of 20° (left) and 60° (right).

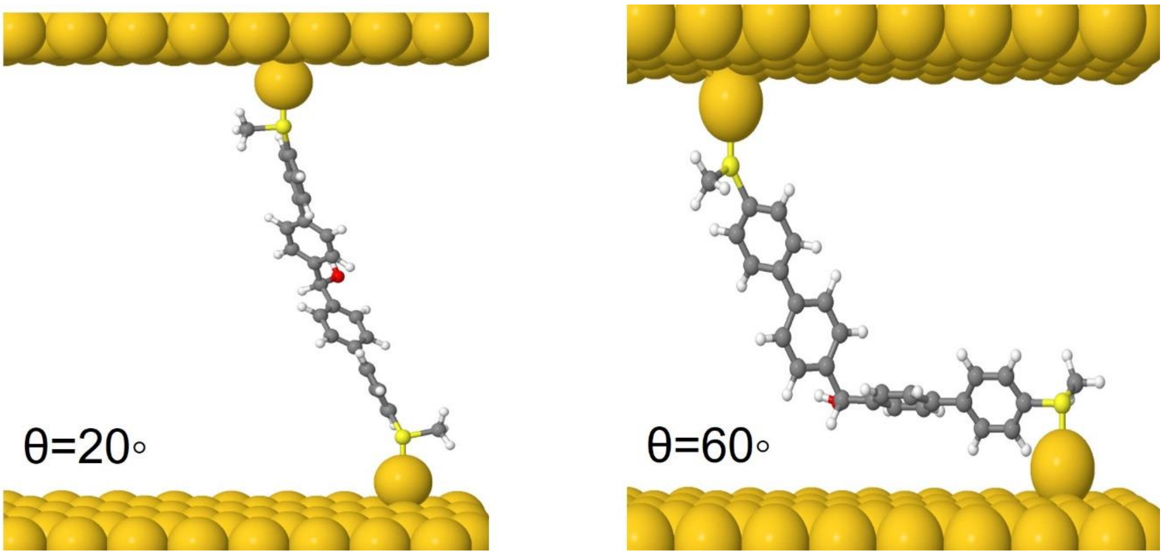

Figure S31. Junction geometry for molecule **3b** connected to gold electrodes *via* a gold adatom for a tilt angle of 20° (left) and 60° (right).

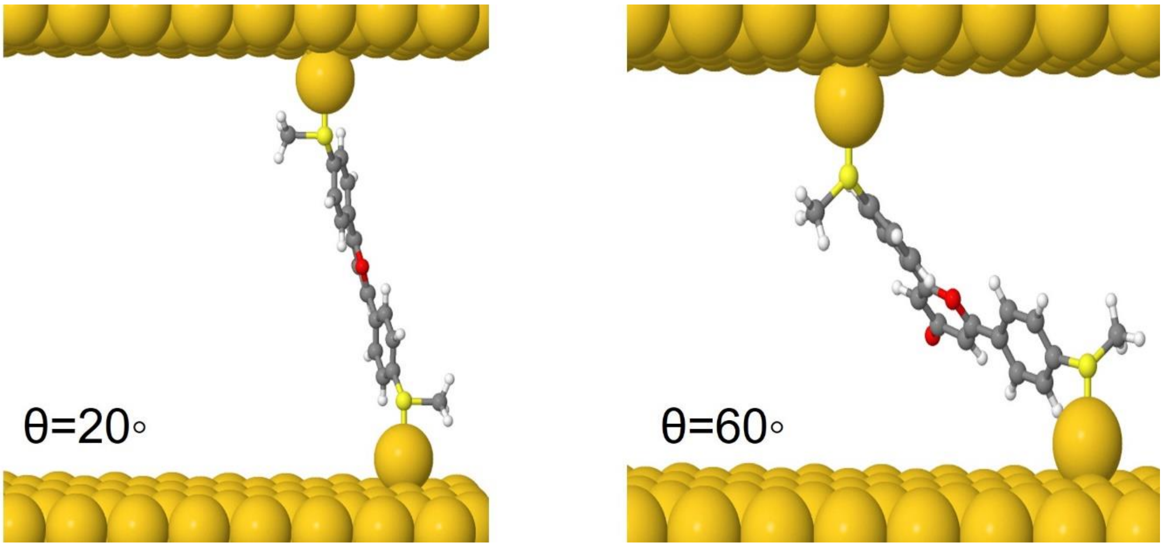

Figure S32. Junction geometry for molecule **4** connected to gold electrodes *via* a gold adatom for a tilt angle of 20° (left) and 60° (right).

S10. Seebeck Coefficient Calculations

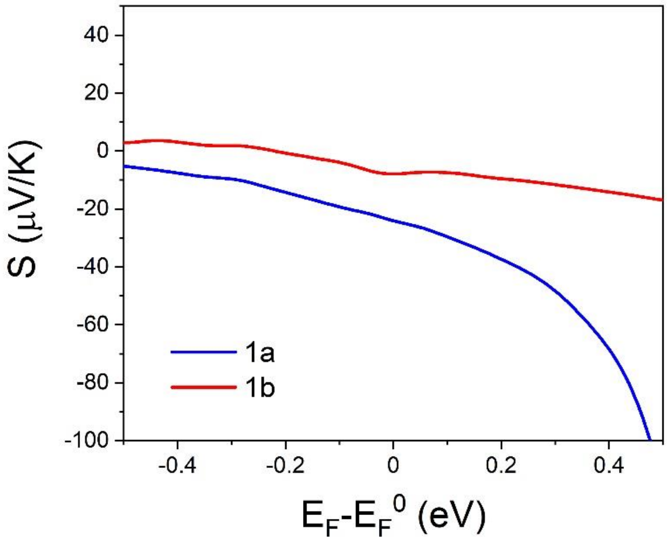

Figure S33. Seebeck coefficient versus Fermi energy for molecules **1a** and **1b** for a tilt angle θ=60°.

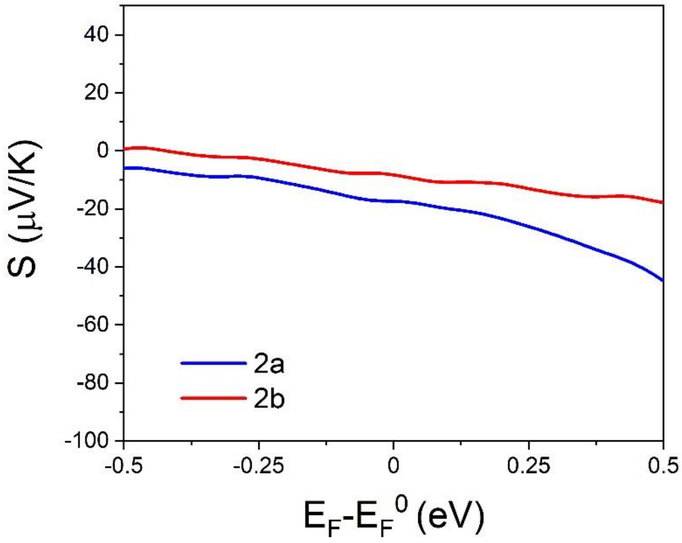

Figure S34. Seebeck coefficient versus Fermi energy for molecules **2a** and **2b** for a tilt angle θ=60°.

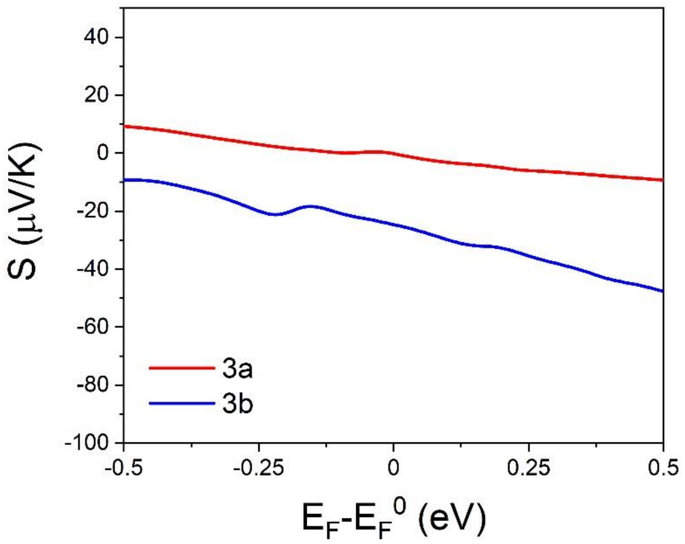

Figure S35. Seebeck coefficient versus Fermi energy for molecules **3a** and **3b** for a tilt angle θ=60°.

S11. Molecule 4 transport calculations

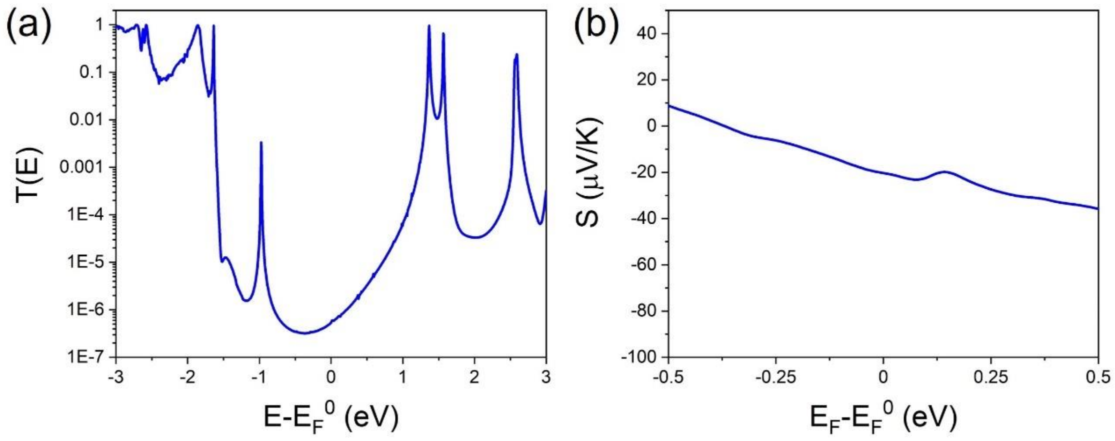

Figure S36. (a) Zero bias transmission coefficient T(E) and (b) Seebeck coefficient for molecule **4** with a tilt angle θ=60°.

S12. Energy of molecular junctions

The total energy of the molecular junction is calculated using DFT for each of the measured molecules for the parameters θ and ϕ that control the junction geometry (Figure S37). The molecular axis is defined as the line between the two terminating sulfur atoms and the tilt angle θ is the angle between this axis and the normal of the gold surface. The angle φ is the rotation of the molecule about the axis defined by the sulfur atoms. Here, the values of θ are varied between 20 and 60° and the value of φ is changed from 0 to 360° to complete a full rotation. The total energy values are shifted relative to the lowest energy configuration which is defined to be 0 eV.

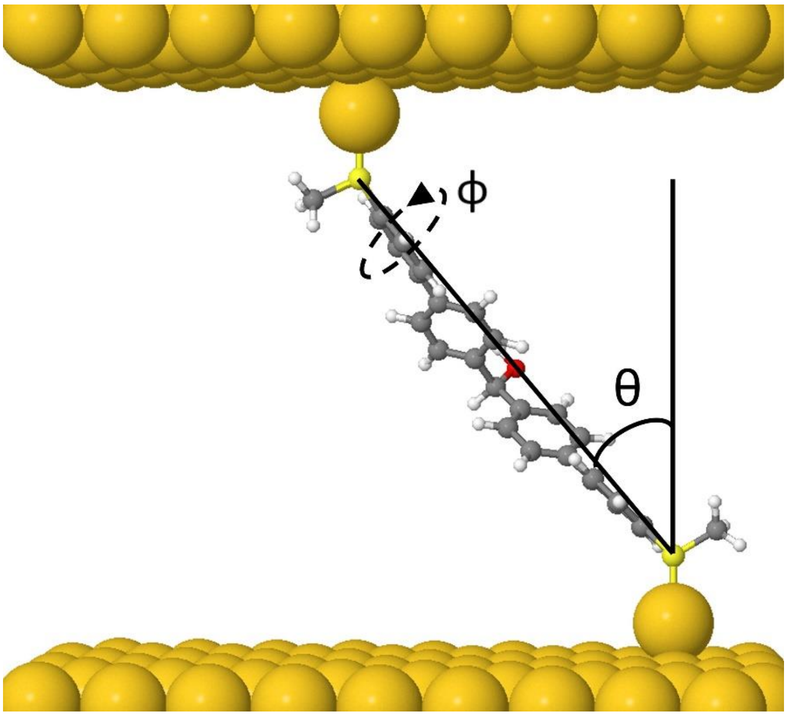

Figure S37. Definition of the molecule geometry within the junction defined by the tilt angle θ and the rotation about the molecule axis ϕ.

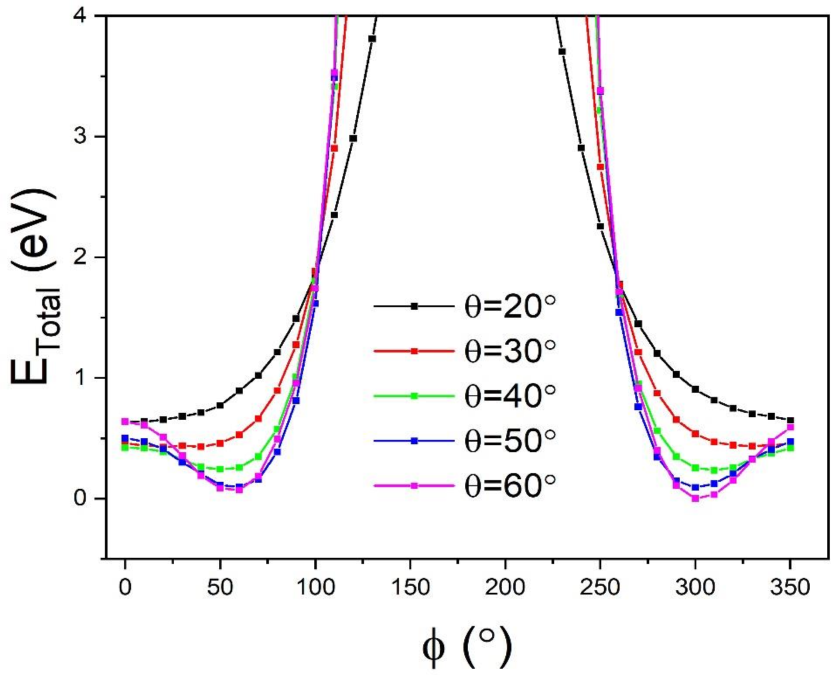

Figure S38. Total energy of **1b** in the junction for parameters θ and ϕ.

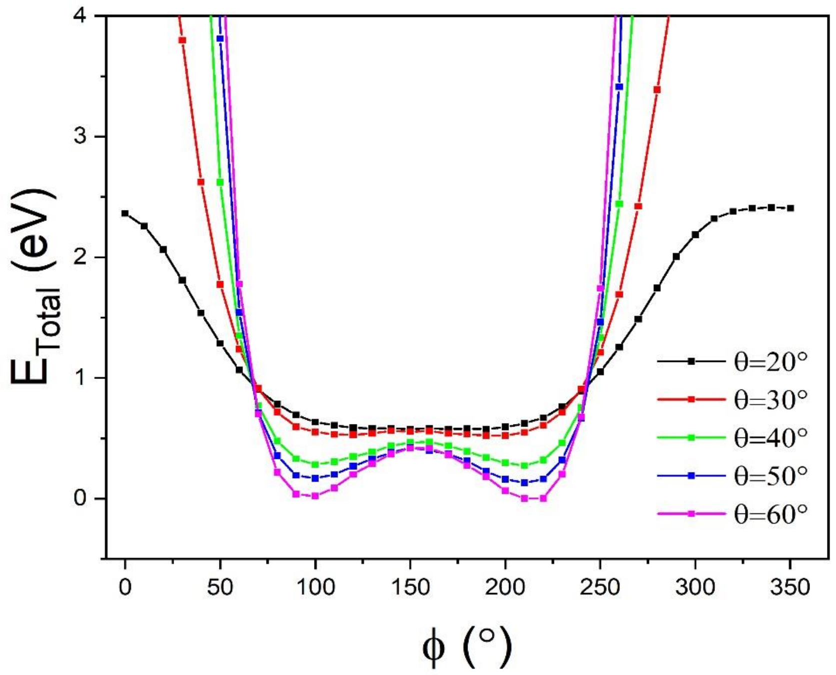

Figure S39. Total energy of **2a** in the junction for parameters θ and ϕ.

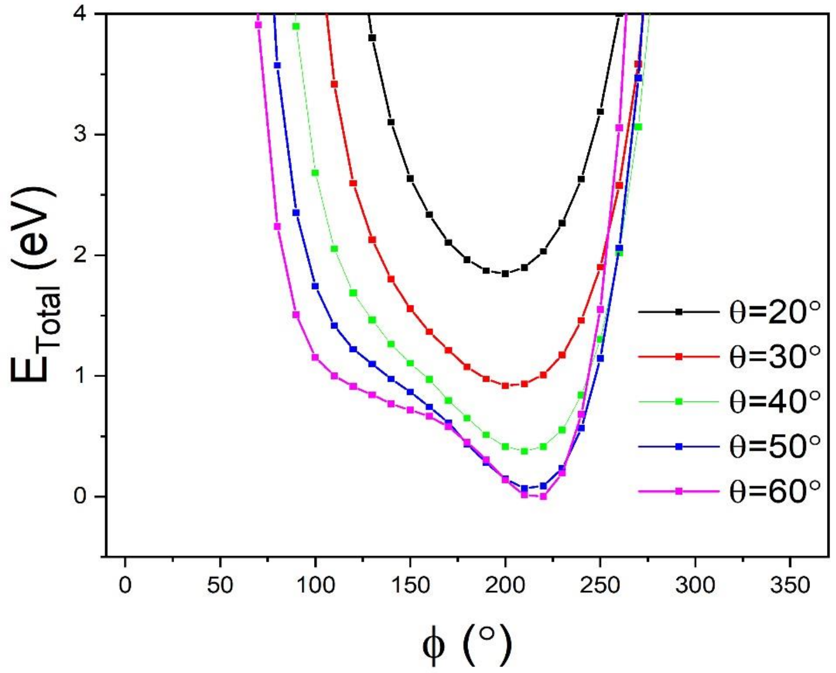

Figure S40. Total energy of **2b** in the junction for parameters θ and ϕ.

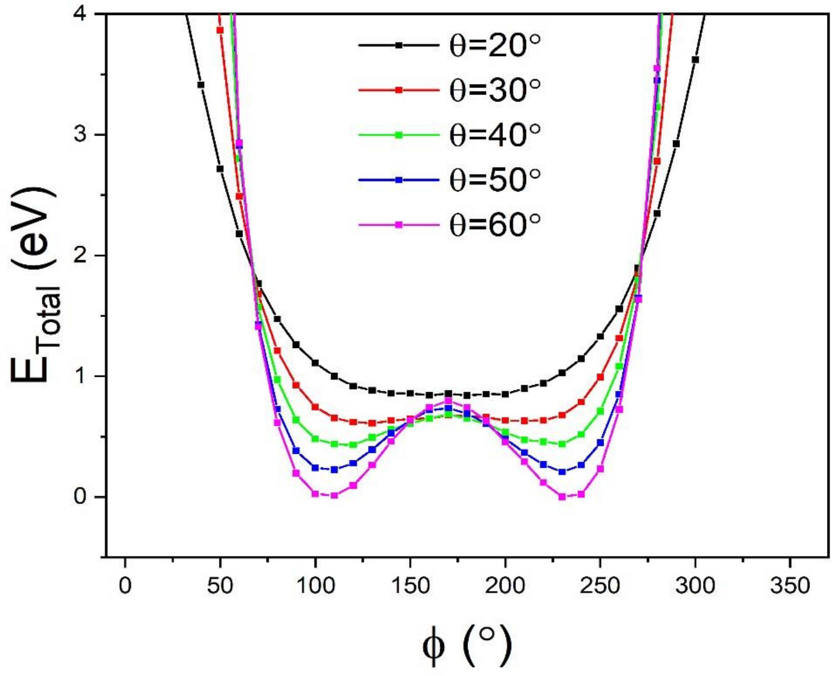

Figure S41. Total energy of **3a** in the junction for parameters θ and ϕ.

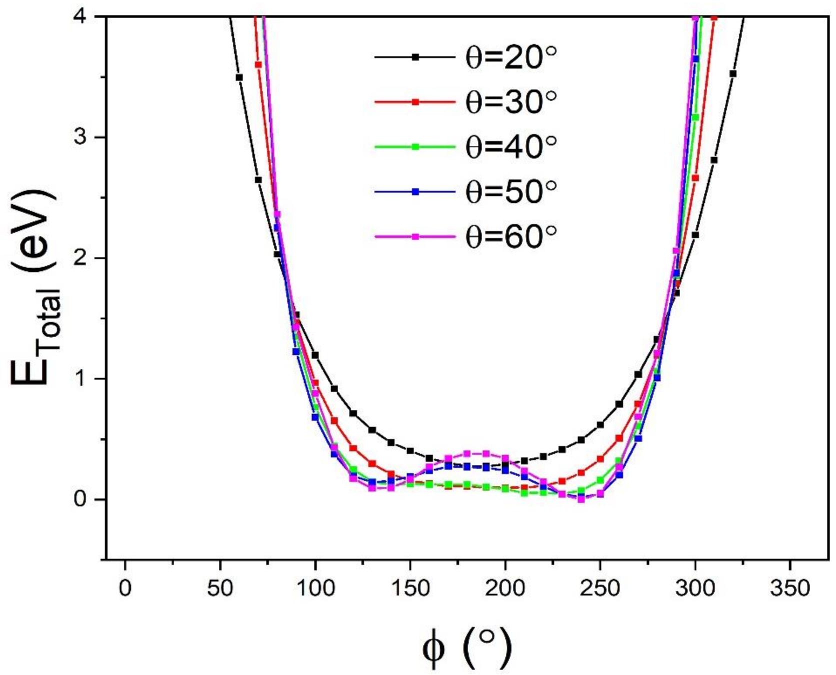

Figure S42. Total energy of **3b** in the junction for parameters θ and ϕ.

These results show that the energy difference between the optimum geometry of θ=60° and θ=20° is large for all the molecules (**1b**= 0.6 eV, **2a**=0.27 eV, **2b**=1.8 eV, **3a**=0.8 eV and **3b**=0.27 eV). However, the energy plots here show that there will be only very small variations in the tilt angle, because the energy barrier for θ between 50° and 60° is typically larger than k_BT at room temperature (**1b**=0.1 eV, **2a**=0.13 eV, **2b** = 0.1 eV, **3a**=0.21 eV and **3b**=0.03 eV). We also calculate the transmission curves to show the comparison between the tilt angle of θ=60° and θ=50°. These show that in almost all cases, there is only a slight variation close to the Fermi energy, so a Boltzmann average is not needed.

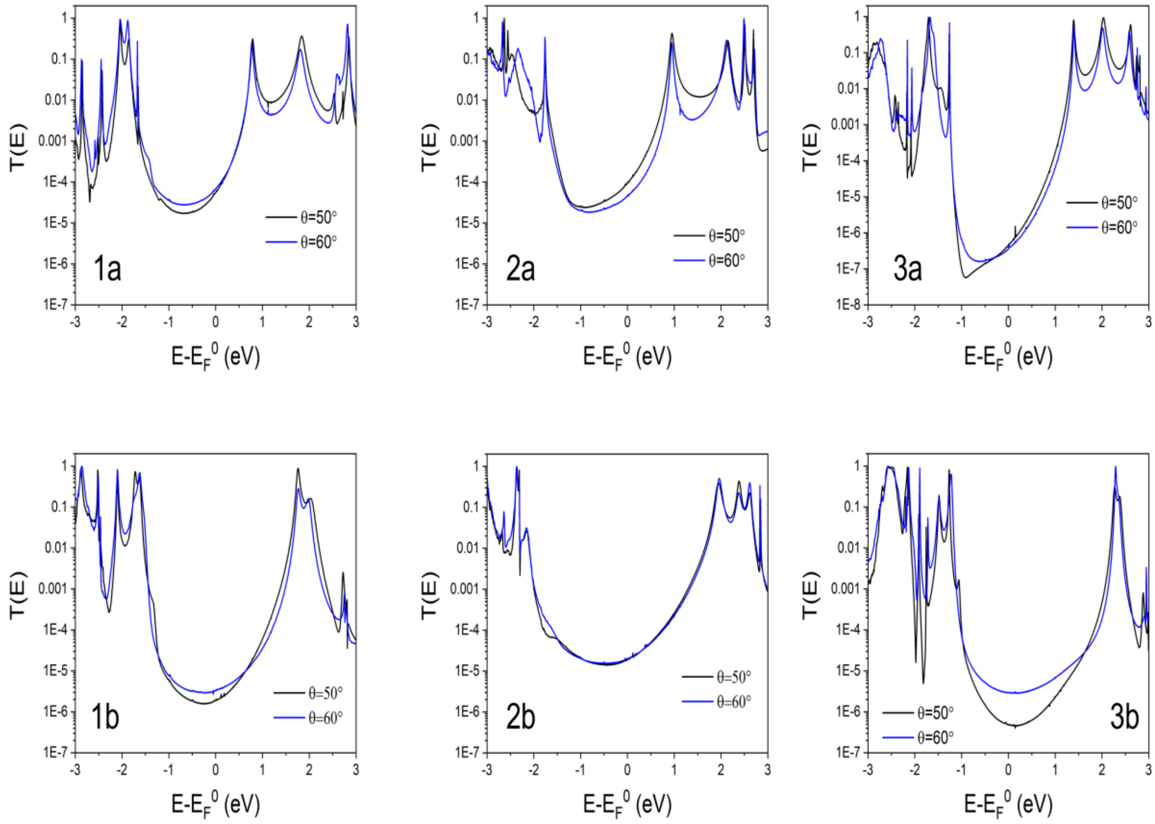

Figure S43. Zero bias transmission coefficient T(E) comparing θ=50° and θ=60° for molecules **1-3**.

References

(1) Cheng, S.; Xin, Y.; Hu, J.; Feng, W.; Ahn, D.; Zeller, M.; He, J.; Xu, Z. Invisible Silver Guests Boost Order in a Framework That Cyclizes and Deposits Ag_3Sb Nanodots. *Inorg. Chem.* **2021**, *60*, 5757–5763.

(2) Chen, L.; Zhang, C.; Lin, G.; Nie, H.; Luo, W.; Zhuang, Z.; Ding, S.; Hu, R.; Su, S. J.; Huang, F. et al. Solution-Processable, Star-Shaped Bipolar Tetraphenylethene Derivatives for the Fabrication of Efficient Nondoped OLEDs. *J. Mater. Chem. C* **2016**, *4*, 2775–2783.

(3) Okunola-Bakare, O. M.; Cao, J.; Kopajtic, T.; Katz, J. L.; Loland, C. J.; Shi, L.; Newman, A. H. Elucidation of Structural Elements for Selectivity Across Monoamine

Transporters: Novel 2-[(Diphenylmethyl)Sulfinyl]Acetamide (Modafinil) Analogues. *J. Med. Chem.* **2014**, *57*, 1000–1013.

(4) Miao, X.; Cai, Z.; Zou, H.; Li, J.; Zhang, S.; Ying, L.; Deng, W. Achieving Halogen Bonding Enhanced Ultra-Highly Efficient AIE and Reversible Mechanochromism Properties of TPE-Based Luminogens: Position of Bromine Substituents. *J. Mater. Chem. C* **2022**, *10*, 8390–8399.

(5) Zhang, L. Y.; Duan, P.; Wang, J. Y.; Zhang, Q. C.; Chen, Z. N. Ruthenium(II) as Conductive Promoter to Alleviate Conductance Attenuation in Oligoynyl Chains. *J. Phys. Chem. C* **2019**, *123*, 5282–5288.

(6) Delcaillau, T.; Boehm, P.; Morandi, B. Nickel-Catalyzed Reversible Functional Group Metathesis between Aryl Nitriles and Aryl Thioethers. *J. Am. Chem. Soc.* **2021**, *143*, 3723–3728.

(7) Su, T. A.; Widawsky, J. R.; Li, H.; Klausen, R. S.; Leighton, J. L.; Steigerwald, M. L.; Venkataraman, L.; Nuckolls, C. Silicon Ring Strain Creates High-Conductance Pathways in Single-Molecule Circuits. *J. Am. Chem. Soc.* **2013**, *135*, 18331–18334.

(8) Qiu, Y. F.; Yang, F.; Qiu, Z. H.; Zhong, M. J.; Wang, L. J.; Ye, Y. Y.; Song, B.; Liang, Y. M. Brønsted Acid Catalyzed and NIS-Promoted Cyclization of Diynones: Selective Synthesis of 4-Pyrone, 4-Pyridone, and 3-Pyrrolone Derivatives. *J. Org. Chem.* **2013**, *78*, 12018–12028.

(9) Krause, L.; Herbst-Irmer, R.; Sheldrick, G. M.; Stalke, D. Comparison of Silver and Molybdenum Microfocus X-Ray Sources for Single-Crystal Structure Determination. *J. Appl. Crystallogr.* **2015**, *48*, 3–10.

(10) Sheldrick, G. M. SHELXT - Integrated Space-Group and Crystal-Structure Determination. *Acta Crystallogr. Sect. A Found. Crystallogr.* **2015**, *71*, 3–8.

(11) Dolomanov, O. V; Bourhis, L. J.; Gildea, R. J.; Howard, J. A. K.; Puschmann, H. OLEX2 : A Complete Structure Solution , Refinement and Analysis Program. *J. Appl. Crystallogr.* **2009**, *42*, 339–341.

(12) Arroyo, C. R.; Leary, E.; Castellanos-Gómez, A.; Rubio-Bollinger, G.; González, M. T.; Agraït, N. Influence of Binding Groups on Molecular Junction Formation. *J. Am. Chem. Soc.* **2011**, *133*, 14313–14319.

(13) Soler, J. M.; Artacho, E.; Gale, J. D.; García, A.; Junquera, J.; Ordejón, P.; Sánchez-Portal, D. The SIESTA Method for Ab Initio Order-N Materials Simulation. *J. Phys. Condens. Matter* **2002**, *14*, 2745.

(14) Ferrer, J.; Lambert, C. J.; García-Suárez, V. M.; Manrique, D. Z.; Visontai, D.; Oroszlany, L.; Rodríguez-Ferradás, R.; Grace, I.; Bailey, S. W. D.; Gillemot, K. et al. GOLLUM: A Next-Generation Simulation Tool for Electron, Thermal and Spin Transport. *New J. Phys.* **2014**, *16*, 93029.
